# Supplementary material for: Examining causal pathways between family adversity and soiling: a prospective cohort study
Source: BMJ Paediatr Open. 2025 Dec 12;9(1):e003978. doi: 10.1136/bmjpo-2025-003978 (PMC12719889; doi:10.1136/bmjpo-2025-003978)
Supplement: Author's manuscript [file bmjpo-2025-003978.draft_revisions.pdf]

BMJ Paediatrics Open

BMJ Paediatrics Open is committed to open peer review. As part of this commitment we make the peer review history of every article we publish publicly available.

When an article is published we post the peer reviewers' comments and the authors' responses online. We also post the versions of the paper that were used during peer review. These are the versions that the peer review comments apply to.

The versions of the paper that follow are the versions that were submitted during the peer review process. They are not the versions of record or the final published versions. They should not be cited or distributed as the published version of this manuscript.

BMJ Paediatrics Open is an open access journal and the full, final, typeset and author-corrected version of record of the manuscript is available on our site with no access controls, subscription charges or pay-per-view fees (http://bmjpaedsopen.bmj.com).

If you have any questions on BMJ Paediatrics Open's open peer review process please email info.bmjpo@bmj.com

BMJ Paediatrics Open

Examining causal pathways between family adversity and soiling: a prospective cohort study

Journal:	*BMJ Paediatrics Open*
Manuscript ID	bmjpo-2025-003978
Article Type:	Original research
Date Submitted by the Author:	14-Aug-2025
Complete List of Authors:	Burrows, Kimberley; University of Bristol Medical School, Centre for Academic Child Health, Population Health Sciences<br>Heron, Jon; University of Bristol Medical School, Centre for Academic Mental Health, Population Health Sciences; University of Bristol Medical School, Medical Research Council Integrative Epidemiology Unit, Population Health Sciences<br>Richardson, Davina; Bladder and Bowel UK, The Toilet Training Team<br>Rayner, Juliette; ERIC, The Children's Bowel and Bladder Charity<br>Spray, Chris; Bristol Royal Hospital for Children, Paediatric Gastroenterology Department<br>Joinson, Carol; University of Bristol Medical School, Centre for Academic Child Health, Population Health Sciences
Keywords:	Child Health, Epidemiology, Statistics

SCHOLARONE™
Manuscripts

I, the Submitting Author has the right to grant and does grant on behalf of all authors of the Work (as defined in the below author licence), an exclusive licence and/or a non-exclusive licence for contributions from authors who are: i) UK Crown employees; ii) where BMJ has agreed a CC-BY licence shall apply, and/or iii) in accordance with the terms applicable for US Federal Government officers or employees acting as part of their official duties; on a worldwide, perpetual, irrevocable, royalty-free basis to BMJ Publishing Group Ltd ("BMJ") its licensees and where the relevant Journal is co-owned by BMJ to the co-owners of the Journal, to publish the Work in this journal and any other BMJ products and to exploit all rights, as set out in our licence.

The Submitting Author accepts and understands that any supply made under these terms is made by BMJ to the Submitting Author unless you are acting as an employee on behalf of your employer or a postgraduate student of an affiliated institution which is paying any applicable article publishing charge ("APC") for Open Access articles. Where the Submitting Author wishes to make the Work available on an Open Access basis (and intends to pay the relevant APC), the terms of reuse of such Open Access shall be governed by a Creative Commons licence – details of these licences and which Creative Commons licence will apply to this Work are set out in our licence referred to above.

Other than as permitted in any relevant BMJ Author's Self Archiving Policies, I confirm this Work has not been accepted for publication elsewhere, is not being considered for publication elsewhere and does not duplicate material already published. I confirm all authors consent to publication of this Work and authorise the granting of this licence.

Confidential: For Review Only

TITLE

Examining causal pathways between family adversity and soiling: a prospective cohort study

AUTHORS

Kimberley Burrows PhD[1], Jon Heron PhD[2,3], Davina Richardson[4], Christine Spray[5], Juliette Rayner[6], Carol Joinson PhD [1]

INSTITUTIONS

1. Centre for Academic Child Health, Population Health Sciences, Bristol Medical School, at the University of Bristol, Bristol, United Kingdom.
2. Centre for Academic Mental Health, Population Health Sciences, Bristol Medical School, at the University of Bristol, Bristol, United Kingdom.
3. Medical Research Council Integrative Epidemiology Unit, Population Health Sciences, Bristol Medical School, at the University of Bristol, Bristol, United Kingdom.
4. Children's Nurse Specialist, Bladder and Bowel UK, The Toilet Training Team.
5. Paediatric Gastroenterology Department, Bristol Royal Hospital for Children, Bristol BS2 8BJ, United Kingdom.
6. ERIC, The Children's Bowel and Bladder Charity, 36 Old School House, Kingswood Foundation, Brittania Rd, Bristol, BS15 8DB, United Kingdom.

Corresponding author: Kimberley Burrows, Centre for Academic Child Health, Population Health Sciences, Bristol Medical School, University of Bristol, Canynge Hall, 39 Whatley Road, Bristol BS8 2PS. Email address: Kimberley.Burrows@bristol.ac.uk

Confidential: For Review Only

Key words: ALSPAC; Cohort Studies; Mediation Analysis; gformula, Constipation, Faecal Incontinence, Adversity

Word count: 2990

Confidential: For Review Only

Abstract [249/300]

Objective: We aimed to examine the associations between family adversity in infancy with subsequent daytime soiling at primary school age. We also examined factors on the casual pathway between family adversity and soiling, specifically the child's emotional/behaviour problems and constipation.

Design: This prospective cohort study included children from the Avon Longitudinal Study of Parents and Children (*N*=10 033) with data on the Family Adversity Index (FAI; birth to age 2), daytime soiling at age 7.5, emotional/behaviour problems at age 4, and constipation at age 6. We examined the relationships between the FAI, soiling, emotional/behaviour problems, and constipation. We then evaluated the mediating effects of emotional/behaviour problems and constipation. All analyses were adjusted for child and family related confounders.

Results: Daytime soiling occurred in 7% of children. A one-unit increase in infancy FAI was associated with increased odds of 12% for daytime soiling at age 7.5 (odds ratio [OR] 1.12, 95% confidence interval [CI] 1.07, 1.17), emotional/behaviour problems score at age 4 (beta 0.52, 95%CI 0.47, 0.58) and increased odds of constipation at age 6 (OR 1.07, 95%CI 1.03, 1.11). The presence of constipation at age 6 was strongly associated with increased odds of soiling at age 7.5 (OR 3.34, 95%CI 2.68, 4.16). There was weak evidence that the associations between FAI and daytime soiling were mediated by emotional/behaviour problems and constipation ($OR_{natural\_indirect\_effect}$ 1.03, 95%CI 0.99-1.07).

Conclusions: Clinicians should be aware that exposure to family adversity in the early years places children at greater risk of subsequent constipation and soiling.

Confidential: For Review Only

Key messages

What is already known on this topic?

- There is growing evidence that adversities including exposure to stressful life events, maternal psychopathology, and family discord may play a role in determining a child's later risk of soiling.

What this study adds?

- The total burden of family adversity (encompassing facets of psychopathology, discord and events) during infancy increases the odds of soiling at primary school age.
- There is weak evidence that emotional/behaviour problems and constipation mediates some of the relationship between family adversity and soiling.

How this study might affect research, practice or policy?

- Clinicians should be aware that exposure to family adversity in the early years places children at greater risk of subsequent soiling.
- Increased resources need to be directed at providing support and interventions for families who are experiencing adversities to reduce the impact on children's health and development.

Confidential: For Review Only

INTRODUCTION

Soiling (faecal incontinence) is a common paediatric health problem (estimated prevalence of 4% in primary school-age children) [1], which causes significant distress to children and their parents [2] as well as presenting challenges within educational settings. Most cases of childhood soiling are functional and are a consequence of overflow due to chronic constipation [3]. Stool withholding and toileting refusal, due to early painful experiences of passing hard stools, are primary causes of functional constipation in children [3].

There is growing evidence that adversity in the child's early family environment plays a role in determining a child's later risk of soiling. Previous studies have focused on specific types of adversities including exposure to stressful life events, maternal psychopathology, and family discord [4–9], but none have examined the total burden of exposure to a range of adversities in the early years and many were cross-sectional or used a retrospective design (reviewed in [5,9]). There is also a lack of research examining mechanisms that could explain how early adverse exposures could increase a child's subsequent risk of soiling.

The current paper, based on data from a large UK birth cohort, examines the prospective relationship between exposure to family adversity in infancy (0-2 years) and subsequent soiling at primary school age (7.5 years). We use a measure of family adversity which comprises multiple domains including deprivation, low parental education, parental discord, parental crime, parental psychopathology, and parents' lack of social/practical/emotional support. We also examine factors on the causal pathway between family adversity and soiling, specifically the child's emotional/behaviour problems and constipation. We chose to focus on these factors

Confidential: For Review Only

because these are potentially modifiable and could be targeted in interventions aimed at reducing the risk of soiling [7,15–19].

METHODS

We used the Strengthening the Reporting of Observational Studies in Epidemiology (STROBE) cohort reporting guidelines (see STROBE checklist in the Supplementary Text).

Sample

ALSPAC is a prospective, population-based birth cohort study that recruited pregnant women resident in Avon, UK with expected dates of delivery between 1st April 1991 and 31st December 1992 [20,21]. The initial number of pregnancies enrolled was 14 541 resulting in 14 062 live births and 13 988 children who were alive at 1 year of age. Further details are given in the Supplementary Text.

The eligible sample for this study included children who had available data on the family adversity index score (FAI; see below) derived for the period between birth and two years of age **(**N = 10 033). Complete data for the FAI, mediators, daytime soiling, and confounders were available for 3366 children. The participant flowchart is presented in Figure 1.

Patient and public involvement

Patients and/or the public were not involved in the design, conduct, reporting or dissemination of this research.

Exposure: family adversity index

Confidential: For Review Only

Multiple family, parental, sociodemographic, and social support indicators were assessed via questionnaires completed during pregnancy and when the child was 0-2 years old. The FAI consisted of 18 items about the age of the mother at pregnancy, housing, parents' education, financial difficulties, relationship status and support, family size and care, social network, substance abuse, crime, and psychopathology of parents. Total FAI score was derived by summing all 18 items, with higher scores indicating greater family adversity. See Supplementary Text and Supplementary Table 1 for further information on the FAI.

Outcomes: daytime soiling

When the study children were 7 years 8 months (SD = 0.14) (hereafter referred to as 7.5 years) mothers were asked "how often does your child dirty their pants during the day". Options included "Never"; "Occasional accidents but less than once a week"; "About once a week"; "2-5 times a week"; "Nearly every day"; and "More than once a day". We derived a binary variable to indicate any daytime soiling by assigning a value of 0 to "Never" and 1 to all other categories. Further information for coding daytime soiling is given in Supplementary Table 1.

Mediators: emotional/behaviour problems and constipation

When the study child was 4 years (SD = 0.12), mothers completed the Strengths & Difficulties Questionnaire (SDQ) [22], which comprises five subscales (each with five items: hyperactivity, conduct problems, emotional symptoms, peer problems and prosocial behaviour). An emotional/behaviour problems score was calculated from the SDQ using the first four subscales (range: 0 to 40). A high score indicates more emotional/behaviour problems.

Confidential: For Review Only

When the study child was 5 years 10 months (SD = 0.12) (hereafter referred to as 6 years) mothers were asked "Has your child had any constipation in the past 12 months". Options included "Yes and saw a doctor"; "Yes but did not see a doctor"; "No, did not have". We derived a binary variable to indicate constipation by assigning a value of 0 to "No, did not have" and 1 to the other two other categories. Further information for coding the mediators is in Supplementary Table 1.

Baseline and intermediate confounders

We adjusted for baseline confounders (measured during the antenatal period or at birth) of each of the mediating pathways (exposure-outcome, exposure-mediator, and mediator-outcome). Confounders measured during the antenatal period included ethnicity, household social class, marital status, and smoking during pregnancy. The confounders sex assigned at birth and preterm birth were measured during the postnatal period. Other indicators of socioeconomic status and maternal psychopathology were intrinsically linked to the factors included in the FAI (either directly or measured very closely in time to those included in the FAI) and were omitted as confounders.

We also included intermediate confounders in our models i.e. factors that may be on the causal pathway from the exposure (FAI) to the mediators (emotional/behaviour problems and constipation) (see Figure 2). These included child developmental delay, maternal bonding score, parental abuse, stool frequency, hard stools, difficult temperament, picky eating, healthy eating, temper tantrums, and BMI all measured between ages 30 to 45 months [7,15–19]. Figure 2Details on the measurement and coding of the confounders and intermediate confounders are given in Supplementary Table 1.

Confidential: For Review Only

Statistical analysis

We used univariable and multivariable logistic and linear regression to examine the relationships between (i) FAI and daytime soiling, (ii) mediators and daytime soiling, and (iii) FAI and mediators (emotional/behaviour problems and constipation).

We used the *gformula* (parametric g-computation formula [23]) command in Stata [24] to examine the mediating effect of emotional/behaviour problems and constipation jointly on the association between FAI score and daytime soiling (see Figure 2). G-formula is based on the counterfactual framework and allows the decomposition of effects into the total causal effect (TCE), the natural indirect effect (NIE) (via the mediators of interest), and the natural direct effect (NDE) (not via the mediators of interest). G-formula can also estimate marginal odds ratios for mediation effects with a binary outcome whilst incorporating intermediate confounders (see Supplementary Text for further information). We used g-formula to decompose the NIE via emotional/behaviour problems and constipation jointly as we did not consider them to be independent. We used a Monte Carlo sample size of 100 000 and we estimated normal-based 95% confidence intervals (CI) using standard errors from 250 bootstrap resamples. The proportion mediated (PM) was calculated as $[OR_{NDE} (OR_{NIE} - 1)] / [OR_{NDE} \times OR_{NIE} - 1] \times 100$ however, this is only interpretable when the TCE, NIE, and NDE have the same direction of effect [25].

Analyses were conducted using Stata version 18 [26] and R version 4.3.0 [27].

Missing data

We used multivariate imputation by chained equations under the missing at random assumption to impute missing data [28] in daytime soiling and baseline and intermediate confounders up to the sample with complete data on the FAI score (N = 10 033). We also included auxiliary variables that are considered predictive of the

Confidential: For Review Only

missing values including socioeconomic status indicators and earlier measures of variables (see Supplementary Table 2). Missing data were imputed separately in males and females before appending the two datasets together. For both males and females 50 imputed datasets with 50 iterations were created using the mice package (version 3.16.0) in R. Estimates were then combined using Rubin's rules [29] (see Supplementary Text).

RESULTS

Table 1 shows the distribution of study variables in the imputed (N = 10 033) and complete case samples (N = 3366). Any daytime soiling was reported for 7.3% of children. In total, 71.7% of children experienced one or more family adversities with the mean number of family adversities experienced was 1.9 (Standard Error (SE) 0.02) in all children and was higher in those experiencing soiling (2.3, SE 0.10) compared to those without soiling (1.8, SE 0.02; ttest p-value <0.001). Further details of the study sample and variables are given in the Supplementary text (Results: description of sample) and Supplementary Table 3.

Confidential: For Review Only

Table 1 Descriptive statistics of study variables for the imputed sample (N = 10 033) and complete case sample (N = 3366)

Variable	Eligible sample (N =< 10,033)		N Missing from eligible sample (N = 10,033)[1]	Imputed sample (N = 10,033)		Complete case sample (N = 3,366)	
	Mean	SE	N [%]	Mean	SE	Mean	SE
FAI (0-13)[2]	1.86	0.02	NA	1.86	0.02	1.56	0.03
Child development level (Z-score)	0.01	0.01	2285 [22.8]	0.01	0.01	-0.01	0.02
Maternal bonding score (10-33)	27.63	0.04	1701 [17.0]	27.61	0.04	27.72	0.06
Child difficult temperament (5-25)	12.43	0.05	1307 [13.0]	12.45	0.05	12.34	0.07
Healthy diet (principal component score)	-0.01	0.01	1345 [13.4]	-0.01	0.01	-0.04	0.02
BMI (kg/m2)	16.23	0.02	3131 [31.2]	16.23	0.02	16.22	0.02
Emotional/behaviour problems score (0-40)	8.77	0.05	1791 [17.9]	8.85	0.05	8.53	0.08
	%	SE	N [%]	%	SE	%	SE
Childs sex assigned at birth (female)	48.73	0.5	NA	48.73	0.5	49.67	0.86
Maternal smoking during pregnancy							
None	79.06	0.41	121 [1.2]	78.95	0.41	84.46	0.62
Yes, quit early	5.14	0.22		5.16	0.22	3.81	0.34
Yes, throughout	15.81	0.37		15.89	0.37	11.74	0.55
Marital status							
Married	81.42	0.39	155 [1.5]	81.28	0.39	86.69	0.59
Single	13.74	0.35		13.84	0.35	9.63	0.51
Divorced/separated/widowed	4.84	0.22		4.88	0.22	3.68	0.32
Household social class (manual)	16.67	0.38	519 [5.17]	17.27	0.39	12.33	0.57
Preterm birth	4.73	0.21	NA	4.73	0.21	4.43	0.35
Ethnicity (non-white)	3.82	0.19	154 [1.5]	3.88	0.2	2.47	0.27
Stool frequency (low)	4.97	0.23	1368 [13.6]	5.14	0.24	5.53	0.39
Hard stools	27.93	0.48	1214 [12.1]	28.08	0.48	29.11	0.78
Parental abuse	4.57	0.23	1589 [15.8]	5.09	0.26	4.04	0.34
Picky eating	14.79	0.38	1298 [12.9]	14.83	0.37	15.06	0.62

nfidential: For Review Only

Temper tantrums	15.55	0.39	1399 [13.9]	15.98	0.4	14.05	0.6
Constipation at 6 years	10.27	0.35	2488 [24.8]	10.77	0.37	10.49	0.53
Daytime soiling	6.72	0.29	2806 [28.0]	7.3	0.3	7	0.44

FAI family adversity index; BMI body mass index. 50 imputed datasets. The complete case sample comprises individuals with data for all variables described. [1] The total number of missing participants for each variable. NA none missing. [2] The FAI is not normally distributed, the median (IQR) is 1 (0-3).

Confidential: For Review Only

Table 2 presents strong evidence that a one-unit increase in the FAI score was associated with increased odds of 12% for daytime soiling at age 7.5 (adjusted Odds Ratio (OR) 1.12, 95% confidence interval (CI) 1.07, 1.17) after adjustment for confounders. We also found that a one-unit increase in the emotional/behaviour problems score at age 4 was associated with an increase in the odds of daytime soiling at age 7.5 (adjusted OR 1.03, 95% CI 1.002, 1.05). The presence of constipation at age 6 was strongly associated with increased odds of soiling at age 7.5 (adjusted OR 3.34, 95% CI 2.68, 4.16).

Table 2 Results for the associations between family adversity index and mediators with daytime soiling in the imputed data sample (N = 10 033)

Outcome: daytime soiling		Univariable			Multivariable		
		**OR**	**95% CI**	**p-value**	**OR**	**95% CI**	**p-value**
**Exposure**	FAI	1.11	1.06, 1.16	<0.001	1.12	1.07, 1.17	<0.001
	Emotional/behaviour problems[1]	1.07	1.05, 1.09	<0.001	1.03	1.00, 1.05	0.04
	Constipation[1,2]	3.32	2.69, 4.10	<0.001	3.34	2.68, 4.16	<0.001

OR odds ratio; CI confidence interval; FAI family adversity index; all multivariable models adjusted for baseline confounders (sex, ethnicity, preterm birth, household social class, marital status, and smoking during pregnancy). [1] Models additionally adjusted for intermediate confounders (child developmental delay, maternal bonding score, parental abuse, stool frequency, hard stools, difficult temperament, picky eating, healthy eating, temper tantrums, and BMI), and FAI. [2] Model additionally adjusted for emotional/behaviour problems. Analysis performed in 50 imputed datasets. Parameter estimates are based on a one-unit increase in FAI, a one-unit increase in emotional/behaviour problems score, and the comparison between yes vs. no for constipation.

A one-unit increase in the FAI score was associated with an increase in the emotional/behaviour problems score (adjusted beta 0.52, 95% CI 0.47, 0.58) and

increased odds of constipation (adjusted OR 1.07, 95% CI 1.03, 1.11; Supplementary Table 4). There was weak evidence that a one-unit increase in the emotional/behaviour problems score was associated with constipation (adjusted OR 1.01, 95% CI 0.99, 1.03; Supplementary Table 5).

Table 3 shows the TCE, NIE, NDE, and the proportion mediated of the one-unit increase in FAI on daytime soiling jointly through the mediators emotional/behaviour problems and constipation. There was weak evidence of a mediating effect (OR_{NIE} 1.03, 95% CI 0.99, 1.07, PM 27%) however, the effects are imprecise with the 95% CI crossing the null.

As a sensitivity analysis, we repeated all analyses with the complete-case sample (N = 3366; Supplementary Tables 6-8). The direction and magnitude of effects were consistent with the imputed data results.

Table 3 Results for the mediation of family adversity index and daytime soiling via emotional/behaviour problems and constipation in the imputed data sample (N = 10 033)

Outcome	Total causal effect		Natural indirect effect		Natural direct effect		PM%
	OR	95% CI	OR	95% CI	OR	95% CI	
Daytime soiling	1.10	1.04, 1.17	1.03	0.99, 1.07	1.07	1.01, 1.14	27

OR odds ratio; CI confidence interval; PM proportion mediated (%). Mediation models were fitted with emotional/behaviour problems and constipation as the mediators of interest; child developmental delay, maternal bonding score, parental abuse, stool frequency, hard stools, difficult temperament, picky eating, healthy eating, temper tantrums, and BMI were considered intermediate confounders. All paths were adjusted for baseline confounders: sex, ethnicity, preterm birth, household social class, marital status, and smoking during pregnancy. Analysis performed in 50 imputed datasets.

DISCUSSION

We found that children who were exposed to family adversity in the first two years of life were at greater risk of soiling at primary school-age (7.5 years). There was weak evidence that subsequent emotional/behaviour problems and constipation mediated

Confidential: For Review Only

this relationship. Notably, we found that early exposure to family adversity increased the risk of subsequent constipation at age 6. Emotional/behaviour problems at age 4 were weakly associated with constipation at age 6. Consistent with previous studies, we found that the presence of constipation was strongly associated with an increased risk of subsequent soiling.

Strengths and limitations

Major strengths of this study include the use of data from a large birth cohort, the prospective design, the availability of data on a wide range of relevant confounders and intermediate confounders, and the use of causal mediation methods to examine causal pathways between family adversity in early childhood and subsequent risk of soiling at school age.

We did not restrict our analysis to children who met clinical diagnostic criteria for faecal incontinence, therefore, the study findings apply to children with soiling in the community and not just those with the most severe soiling [17]. This is a community-based sample, and therefore, the number of participants who experienced soiling at high frequencies is small compared with clinical samples (0.83%). We therefore examined the presence versus absence of any soiling and did not further categorise by frequency because this would have resulted in small group sizes and lack of precision in our estimates. It is important to note that we found robust associations between family adversity and soiling, even when examining soiling that did not meet the criteria for clinical diagnosis [30]. Parents were not asked about the duration of constipation or soiling at each time point, but the repeated measures of constipation and soiling in the ALSPAC cohort indicate that constipation and soiling are chronic problems [17].

Confidential: For Review Only

The complete-case sample had a lower proportion of participants with socioeconomic disadvantage (e.g. manual social class and divorced/separated/widowed marital status) and FAI than the wider ALSPAC cohort, which may limit the internal validity of our findings. We therefore used multiple imputation to address possible bias due to missing data and report all results as recommended by Sterne et al. [31].

Reliance on parental reports of constipation is a possible limitation. Parents were asked to report if their child had suffered from constipation in the past 12 months and if they had seen doctor but were not asked about the frequency or severity of constipation. Seeing a doctor for constipation is not necessarily an indicator of severity but might be determined by factors such as parental health literacy. Underreporting of constipation is possible since some parents may have been unaware that their child is constipated. However, the fact that the prevalence of constipation in ALSPAC is similar to other population-based studies [32] provides reassurance concerning the validity of the parental reports. The use of parental reports of soiling and the specific phrasing of the question ("how often does your child dirty their pants during the day") could have led to the possibility of parents responding 'yes' to the soiling question due to their child's inadequate wiping following a bowel movement (rather than an episode of faecal incontinence). It is notable, however, that some parents stated in the questionnaire that their child had soiled his/her pants only because of poor wiping or due to suffering an episode of diarrhoea and in these cases they did not place their child in the soiling category [7].

We did not differentiate between retentive and non-retentive subtypes of soiling. Clinical studies, however, estimate that over 80% of children who experience functional faecal incontinence have underlying constipation [30]. We also did not

Confidential: For Review Only

examine nocturnal soiling as an outcome at age 7.5, but the prevalence was very low at this age in the ALSPAC cohort (0.8%).

Comparison with previous findings and potential mechanisms

We found strong evidence that early exposure to family adversity increased the risk of subsequent soiling and constipation. Family-related stressors have previously been linked to constipation in cross-sectional studies [6,33]. There is evidence of the effect of stress on bowel functioning through the gut-brain axis; a bidirectional communication system including the neural, endocrine, microbial, and immune system that connects the two [34]. For example, modulation of bowel motility, secretion, and mucosal responses are controlled by the brain [35]. Development of the gut-brain axis during early infancy and childhood may be impacted by psychosocial factors including stressful events and adversity [34] and may be a plausible mechanism through which early family adversity increases risk of later constipation and soiling.

We found only weak evidence for the association between emotional/behavioural problems score at age 4 and constipation at age 6. This is contrary to a previous study using ALSPAC data, which reported strong evidence of associations between emotional/behavioural problems at 3.5 years and trajectories of constipation and soiling from age 4-10 years [7]. However, the current study uses a measure of constipation at a single timepoint, compared with the previous study which examined trajectories comprising repeated measures of soiling and constipation across childhood.

We found only weak evidence that emotional/behaviour problems and constipation mediated the relationship between family adversity and children's soiling. It is possible that family adversity is linked to an increased risk of childhood soiling through other causal pathways. For example, inadequate toilet training practices have been linked

Confidential: For Review Only

to an increased risk of bowel disorders [36]. ALSPAC did not collect data on the strategies employed by parents during toilet training, but did collect data on age at initiation of toilet training. However, there was little evidence that age at initiation of toilet training was associated with constipation and/or soiling at primary school age [17]. Further research is needed to examine toilet training practices as possible mediators of the link between family adversity and soiling.

Data were not available on important factors at preschool age that are associated with the development of childhood constipation including stool withholding and toileting refusal. However, our analysis did include hard stools and low stool frequency at age 2½ years as intermediate confounders. These problems can be both causes and consequences of stool withholding and toileting refusal. It has previously been argued that stool withholding is unlikely to be caused primarily by emotional/behaviour problems, since painful defecation usually precedes the onset of stool toileting refusal [14]. However, earlier studies based on small clinic samples have found evidence for more difficult temperament traits in children who are difficult to toilet train, and in children with stool toileting refusal [12,13]. There is also evidence that autistic traits and ADHD are associated with soiling, and it is suggested that this could be due to sensory issues which lead to stool withholding and constipation [37,38].

At primary school-age, the focus of this study, other causes of stool withholding include limited access to toilets, hygiene concerns, and perceived lack of privacy or safety of school toilets [39]. Further research is needed to determine the causes of stool toileting refusal in children at different ages.

Implications

Clinicians should be aware that exposure to family adversity in the early years places children at greater risk of subsequent constipation and soiling. Screening for

Confidential: For Review Only

adversities could be conducted at routine contacts with health visitors, GPs, and in paediatric bladder and bowel clinics. Increased resources need to be directed at providing support and interventions for families who are experiencing adversities to reduce the impact on children's health and development.

Constipation in preschool children is still not universally understood and managed by primary care practitioners in much of the UK, despite NICE guidance and a wide evidence base for proactive treatment [40]. There are still some areas in the UK without bladder and bowel services for children and other areas where services do exist but are restricted to seeing children aged over 5 years or are inadequately resourced resulting in long wait times for children to receive specialist assessment and support. This is a serious concern because chronic constipation and soiling in children have adverse impacts on their quality of life and psychosocial functioning [40]. Our findings confirm the importance of training for all health care professionals, early years practitioners and parents/carers on signs and symptoms of constipation in children from birth onwards to enable prompt intervention before it becomes chronic [41].

Confidential: For Review Only

CONTRIBUTORS

CJ, JH, KB: conceptualisation; CJ, JH: funding acquisition; KB, CJ, JH: analytical plan; KB, JH, CJ: methodology; KB: formal analysis; KB, CJ, JH, DR, JR, CS: interpretation of findings; KB, CJ: writing original draft; KB, CJ, JH, DR, JR, CS: writing review and editing.

COMPETING INTERESTS

None declared

FUNDING

This work is supported by funding from the Medical Research Council [grant ref: MR/V033581/1: Mental Health and Incontinence]. JH is a member of the MRC Integrative Epidemiology Unit at the University of Bristol [MC_UU_00011/7]. The UK Medical Research Council and Wellcome [grant ref: 217065/Z/19/Z] and the University of Bristol provide core support for ALSPAC. This publication is the work of the authors and Kimberley Burrows will serve as guarantors for the contents of this paper. A comprehensive list of grants funding is available on the ALSPAC website: http://www.bristol.ac.uk/alspac/external/documents/grant-acknowledgements.pdf.

ACKNOWLEDGEMENTS

We are extremely grateful to all the families who took part in this study, the midwives for their help in recruiting them, and the whole ALSPAC team, which includes interviewers, computer and laboratory technicians, clerical workers, research scientists, volunteers, managers, receptionists, and nurses.

DATA AVAILABILITY

Confidential: For Review Only

The ALSPAC data underlying this article are available upon request from the ALSPAC Executive Committee for researchers who meet the criteria for access to confidential data (https://bristol.ac.uk/alspac/researchers/access/). Code to conduct these analyses can be found on https://github.com/burrowsk/Family-adversity-constipation-soiling.

Ethics approval

Ethical approval for the study was obtained from the ALSPAC Ethics and Law Committee and the Local Research Ethics Committees. Informed consent for the use of data collected via questionnaires and clinics was obtained from participants following the recommendations of the ALSPAC Ethics and Law Committee at the time.

SUPPLEMENTAL MATERIAL

This content has been supplied by the author(s). It has not been vetted by BMJ Publishing Group Limited (BMJ) and may not have been peer-reviewed. Any opinions or recommendations discussed are solely those of the author(s) and are not endorsed by BMJ. BMJ disclaims all liability and responsibility arising from any reliance placed on the content. Where the content includes any translated material, BMJ does not warrant the accuracy and reliability of the translations (including but not limited to local regulations, clinical guidelines, terminology, drug names and drug dosages), and is not responsible for any error and/or omissions arising from translation and adaptation or otherwise.

Confidential: For Review Only

REFERENCES

[1] van der Wal MF, Benninga MA, Hirasing RA. The prevalence of encopresis in a multicultural population. J Pediatr Gastroenterol Nutr 2005;40:345–8. https://doi.org/10.1097/01.mpg.0000149964.77418.27.
[2] Kovacic K, Sood MR, Mugie S, et al. A multicenter study on childhood constipation and fecal incontinence: effects on quality of life. J Pediatr 2015;166:1482-1487.e1. https://doi.org/10.1016/j.jpeds.2015.03.016.
[3] Koppen IJN, Benninga MA. Functional Constipation and Dyssynergic Defecation in Children. Front Pediatr 2022;10:832877. https://doi.org/10.3389/fped.2022.832877.
[4] Chung JM, Lee SD, Kang DI, et al. An epidemiologic study of voiding and bowel habits in Korean children: a nationwide multicenter study. Urology 2010;76:215–9. https://doi.org/10.1016/j.urology.2009.12.022.
[5] Philips EM, Peeters B, Teeuw AH, et al. Stressful Life Events in Children With Functional Defecation Disorders. J Pediatr Gastroenterol Nutr 2015;61:384–92. https://doi.org/10.1097/MPG.0000000000000882.
[6] Oswari H, Alatas FS, Hegar B, et al. Epidemiology of Paediatric constipation in Indonesia and its association with exposure to stressful life events. BMC Gastroenterology 2018;18:146. https://doi.org/10.1186/s12876-018-0873-0.
[7] Joinson C, Grzeda MT, von Gontard A, et al. Psychosocial risks for constipation and soiling in primary school children. Eur Child Adolesc Psychiatry 2019;28:203–10. https://doi.org/10.1007/s00787-018-1162-8.
[8] Sawyer G, Heron J, Joinson C. The relationship between maternal psychopathology and offspring incontinence and constipation at school age: A prospective cohort study. J Affect Disord 2023;335:1–9. https://doi.org/10.1016/j.jad.2023.05.003.
[9] Gozali FS, Febiana B, Putra IGNS, et al. Relationship between psychological stress with functional constipation in children: a systematic review. Pan Afr Med J 2023;46:8. https://doi.org/10.11604/pamj.2023.46.8.41130.
[10] Ahmad T, Banez GA, Steffen RM, et al. DEFECATION ANXIETY IN CHILDREN WITH FUNCTIONAL CONSTIPATION: 804. Official Journal of the American College of Gastroenterology | ACG 2003;98:S267. https://doi.org/10.1111/j.1572-0241.2003.08543.x.
[11] Scully C, McLaughlin J, Fitzgerald A. The relationship between adverse childhood experiences, family functioning, and mental health problems among children and adolescents: a systematic review. Journal of Family Therapy 2020;42:291–316. https://doi.org/10.1111/1467-6427.12263.
[12] Blum NJ, Taubman B, Osborne ML. Behavioral characteristics of children with stool toileting refusal. Pediatrics 1997;99:50–3. https://doi.org/10.1542/peds.99.1.50.
[13] Schonwald A, Sherritt L, Stadtler A, et al. Factors associated with difficult toilet training. Pediatrics 2004;113:1753–7. https://doi.org/10.1542/peds.113.6.1753.
[14] Malowitz S, Green M, Karpinski A, et al. Age of Onset of Functional Constipation. J Pediatr Gastroenterol Nutr 2016;62:600–2. https://doi.org/10.1097/MPG.0000000000001011.
[15] van Dijk M, de Vries G-J, Last BF, et al. Parental child-rearing attitudes are associated with functional constipation in childhood. Arch Dis Child 2015;100:329–33. https://doi.org/10.1136/archdischild-2014-305941.

Confidential: For Review Only

[16] Taylor CM, Northstone K, Wernimont SM, et al. Picky eating in preschool children: Associations with dietary fibre intakes and stool hardness. Appetite 2016;100:263–71. https://doi.org/10.1016/j.appet.2016.02.021.

[17] Heron J, Grzeda M, Tappin D, et al. Early childhood risk factors for constipation and soiling at school age: an observational cohort study. BMJ Paediatr Open 2018;2:e000230. https://doi.org/10.1136/bmjpo-2017-000230.

[18] Tappin D, Grzeda M, Joinson C, et al. Challenging the view that lack of fibre causes childhood constipation. Arch Dis Child 2020;105:864–8. https://doi.org/10.1136/archdischild-2019-318082.

[19] Wang GN, Zhang K, Xiong YY, et al. The relationship between functional constipation and overweight/obesity in children: a systematic review and meta-analysis. Pediatr Res 2023;94:1878–86. https://doi.org/10.1038/s41390-023-02711-1.

[20] Fraser A, Macdonald-Wallis C, Tilling K, et al. Cohort profile: the Avon Longitudinal Study of Parents and Children: ALSPAC mothers cohort. Int J Epidemiol 2013;42:97–110. https://doi.org/10.1093/ije/dys066.

[21] Boyd A, Golding J, Macleod J, et al. Cohort profile: The 'Children of the 90s'—the index offspring of the Avon Longitudinal Study of Parents and Children. Int J Epidemiol 2013;42:111–27. https://doi.org/10.1093/ije/dys064.

[22] Goodman R. Psychometric properties of the strengths and difficulties questionnaire. J Am Acad Child Adolesc Psychiatry 2001;40:1337–45. https://doi.org/10.1097/00004583-200111000-00015.

[23] Robins J. A new approach to causal inference in mortality studies with a sustained exposure period—application to control of the healthy worker survivor effect. Mathematical Modelling 1986;7:1393–512. https://doi.org/10.1016/0270-0255(86)90088-6.

[24] Daniel RM, De Stavola BL, Cousens SN. Gformula: Estimating Causal Effects in the Presence of Time-Varying Confounding or Mediation using the G-Computation Formula. The Stata Journal 2011;11:479–517. https://doi.org/10.1177/1536867X1201100401.

[25] VanderWeele TJ, Vansteelandt S. Odds Ratios for Mediation Analysis for a Dichotomous Outcome. Am J Epidemiol 2010;172:1339–48. https://doi.org/10.1093/aje/kwq332.

[26] StataCorp. Stata Statistical Software: Release 17 2023.

[27] R Core Team. R: A language and environment for statistical computing. 2022.

[28] Royston P, White IR. Multiple Imputation by Chained Equations (MICE): Implementation in Stata. Journal of Statistical Software 2011;45:1–20. https://doi.org/10.18637/jss.v045.i04.

[29] Rubin DB. Multiple Imputation for Nonresponse in Surveys. New York, NY: John Wiley & Sons; 2004.

[30] Koppen IJN, von Gontard A, Chase J, et al. Management of functional nonretentive fecal incontinence in children: Recommendations from the International Children's Continence Society. J Pediatr Urol 2016;12:56–64. https://doi.org/10.1016/j.jpurol.2015.09.008.

[31] Sterne JAC, White IR, Carlin JB, et al. Multiple imputation for missing data in epidemiological and clinical research: potential and pitfalls. BMJ 2009;338:b2393–b2393. https://doi.org/10.1136/bmj.b2393.

[32] Mugie SM, Benninga MA, Di Lorenzo C. Epidemiology of constipation in children and adults: A systematic review. Best Practice & Research Clinical Gastroenterology 2011;25:3–18. https://doi.org/10.1016/j.bpg.2010.12.010.

Confidential: For Review Only

[33] Edan OAA, Yahya FS. Socio-demographic characteristics and risk factors of functional constipation in children: A case-control study. Sri Lanka Journal of Child Health 2022;51. https://doi.org/10.4038/sljch.v51i2.10123.
[34] Jena A, Montoya CA, Mullaney JA, et al. Gut-Brain Axis in the Early Postnatal Years of Life: A Developmental Perspective. Front Integr Neurosci 2020;14. https://doi.org/10.3389/fnint.2020.00044.
[35] Rhee SH, Pothoulakis C, Mayer EA. Principles and clinical implications of the brain-gut-enteric microbiota axis. Nat Rev Gastroenterol Hepatol 2009;6:306–14. https://doi.org/10.1038/nrgastro.2009.35.
[36] Mota DM, Barros AJD. Toilet training: methods, parental expectations and associated dysfunctions. J Pediatr (Rio J) 2008;84:9–17. https://doi.org/10.2223/JPED.1752.
[37] Chogle A, Wong G, Megerian JT. Constipation in Children with Autism: A Comprehensive Review. Curr Treat Options Peds 2024;10:287–94. https://doi.org/10.1007/s40746-024-00304-6.
[38] Gyamenah P, Burrows K, Rai D, et al. Associations of Autistic Traits and Autism with Incontinence and Constipation in a UK Birth Cohort. J Autism Dev Disord 2024. https://doi.org/10.1007/s10803-024-06663-1.
[39] Shkalim Zemer V, Cohen HA, Richenberg Y, et al. Personal hygiene, environmental conditions, and toilet use of children in primary schools: A cohort study. J Pediatr Urol 2023;19:721–7. https://doi.org/10.1016/j.jpurol.2023.06.004.
[40] Vriesman MH, Rajindrajith S, Koppen IJN, et al. Quality of Life in Children with Functional Constipation: A Systematic Review and Meta-Analysis. J Pediatr 2019;214:141–50. https://doi.org/10.1016/j.jpeds.2019.06.059.
[41] England NHS. NHS England » National primary care clinical pathway for constipation in children 2023. https://www.england.nhs.uk/publication/national-primary-care-clinical-pathway-for-constipation-in-children/ (accessed April 3, 2025).

FIGURES

Figure 1 Flow chart of inclusion and exclusion of ALSPAC participants into the study sample

ALSPAC Avon longitudinal study of parents and children; FAI family adversity index. Mediators include emotional/behaviour problems and constipation. Confounders include sex, ethnicity, preterm birth, household social class, marital status, and smoking during pregnancy. Intermediate confounders include child developmental delay, maternal bond score, parental abuse, stool frequency, hard stools, difficult temperament, picky eating, healthy eating, temper tantrums, and BMI.

Figure 2 Directed Acyclic Graph (DAG) illustrating hypothesised causal relationships between the family adversity index, mediators and daytime soiling

Directed acyclic graph (DAG) of the mediation model. Each intermediate confounding path was specified separately but are grouped together for clarity. For simplicity baseline confounders are omitted from the DAG as all confounders are proposed to cause all other model variables.

Confidential: For Review Only

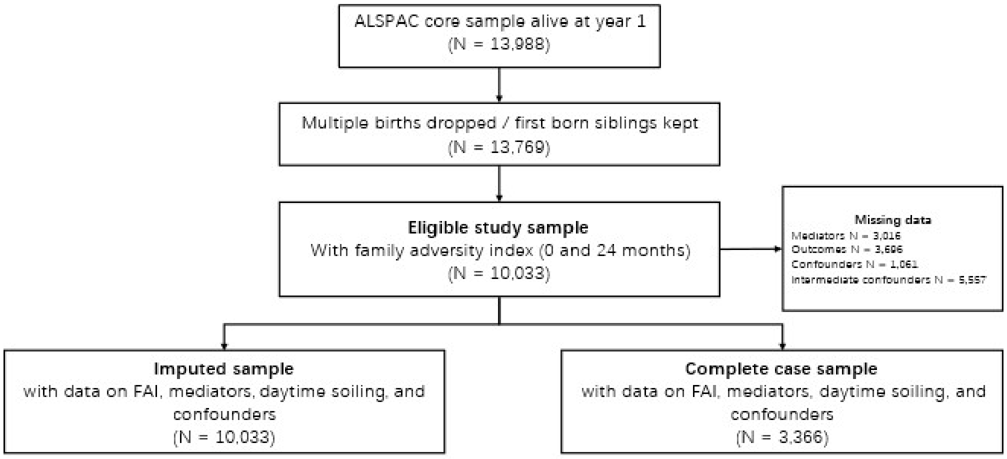

ALSPAC Avon longitudinal study of parents and children; FAI family adversity index. Mediators include emotional/behaviour problems and constipation. Confounders include sex, ethnicity, preterm birth, household social class, marital status, and smoking during pregnancy. Intermediate confounders include child developmental delay, maternal bond score, parental abuse, stool frequency, hard stools, difficult temperament, picky eating, healthy eating, temper tantrums, and BMI.

173x79mm (96 x 96 DPI)

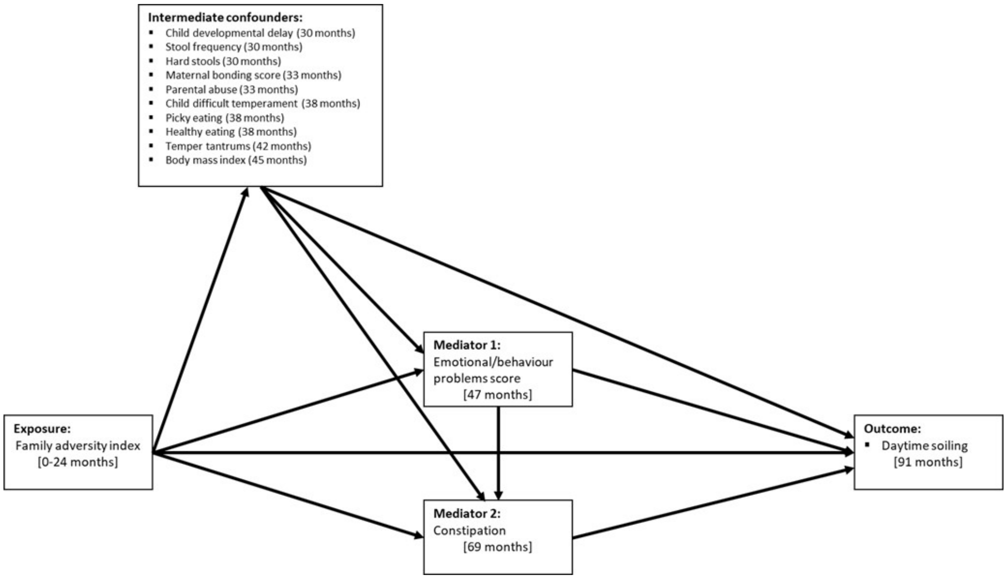

Directed acyclic graph (DAG) of the mediation model. Each intermediate confounding path was specified separately but are grouped together for clarity. For simplicity baseline confounders are omitted from the DAG as all confounders are proposed to cause all other model variables.

174x99mm (150 x 150 DPI)

Examining causal pathways between family adversity and soiling: a prospective cohort study

Contents

Supplementary Text....2
Methods: Sample 2
Methods: The Family Adversity Index (FAI) 2
Methods: The parametric g-formula....3
Methods: Missing data 3
Results: Description of sample....4
Supplementary Tables....5
Table S1 Variable description and coding 5
Table S2 Auxiliary variables for multiple imputation of missing data 10
Table S3 Prevalences of the individual adversity components of the family adversity index in (N = 10,033)....11
Table S4 Results for the associations between family adversity index and mediators in the imputed data sample (N = 10,033)....12
Table S5 Results for the associations between emotional/behaviour problems and constipation in the imputed data sample (N = 10,033)....12
Table S6 Results for the associations between family adversity index and mediators with daytime soiling in the complete case sample (N = 3,366) 12
Table S7 Results for the associations between family adversity index and mediators in the complete case sample (N = 3,366) 13
Table S8 Results for the mediation of family adversity index and daytime soiling via emotional/behaviour problems and constipation in the complete case sample (N = 3,366)....13
References 14
Strobe statement 15

Confidential: For Review Only

Supplementary Text

Methods: Sample

ALSPAC is a prospective, population-based birth cohort study that recruited pregnant women resident in Avon, UK with expected dates of delivery between 1st April 1991 and 31st December 1992 [1,2]. The initial number of pregnancies enrolled was 14,541 resulting in 14,062 live births and 13,988 children who were alive at 1 year of age. When the oldest children were approximately 7 years of age, an attempt was made to bolster the initial sample with eligible cases who had failed to join the study originally, resulting in an additional 913 children being enrolled. The total sample size for analyses using any data collected after the age of seven is therefore 15 447 pregnancies, resulting in 15,658 foetuses. Of these 14,901 children were alive at 1 year of age. 14,203 unique mothers were initially enrolled in the study and 12,113 partners have been in contact with the study of which 3,807 are enrolled. The phases of enrolment are described in more detail in the cohort profile paper and its update [1,2]. The study website (www.bristol.ac.uk/alspac) contains details of all the data that is available through a fully searchable data dictionary and variable search tool: http://www.bristol.ac.uk/alspac/researchers/our-data/. Ethical approval for ALSPAC was obtained from the ALSPAC Ethics and Law Committee and the Local Research Ethics Committees. Informed consent for the use of data collected via questionnaires and clinics was obtained from participants following the recommendations of the ALSPAC Ethics and Law Committee at the time.

Methods: The Family Adversity Index (FAI)

The FAI was derived from responses to questions asked about ALSPAC participants (parents and children) that reflected the following family-based risk factors:

- **Age of mother*** (1): at first pregnancy/child's birth
- **Housing** (3): a) adequacy, b) basic living, c) defects/infestation
- **No educational qualifications*** (1) achievement (mother or father)
- **Financial status** (1): a) financial difficulties
- **Partner relationship** (4): a) status, b) affection and aggression, c) physical/emotional cruelty, d) no social support
- **Family** (2): a) family size, b) Major problems (child in care, not with natural mother, or on Social Services at-risk register**)
- **Social network** (2): a) emotional support, b) Practical/financial support
- **Maternal Psychopathology** (1): a) Affective (depression, anxiety, or suicide attempts)
- **Substance abuse** (1): a) drugs or alcohol (use of hard drugs, alcoholism, alcohol consumption)
- **Crime** (2): a) In trouble with police, or b) convictions

*[The numbers in brackets refer to the number of items within each category. *Note that these items were measured during the antenatal period or at birth. **The at-risk register has been superseded by the Child Protection Plan (CPP); The Children Act 1989]*

The FAI scores were calculated where >50% of the above items were valid and non-adversity was assumed for any missing data. Each item was assigned a value of 1 if the adversity was present and 0 if not. The final score was derived from the sum of

Confidential: For Review Only

items giving a possible score range from 0 to 18 and the higher the score recorded the greater the level of adversity.

Details of the questionnaires from which these items were derived are available in the ALSPAC study documentation file D4297_mLon.pdf held in the publicly available data dictionary:
https://www.bristol.ac.uk/alspac/external/documents/ALSPAC_Data_Dictionary.zip

Methods: The parametric g-formula

In mediation analysis, four assumptions are made with respect to confounding: no unmeasured confounders for the exposure-outcome, exposure-mediator or mediator-outcome paths; no measured or unmeasured confounders of the mediator-outcome path that lie on the causal pathway from the exposure. We implemented the parametric g-computation approach, which relaxes the assumption of no measured intermediate confounders [3,4].

We used the Stata package *gformula* to conduct parametric g-computation to estimate the total causal effect (TCE), natural direct effect (NDE) and the natural indirect effect (NIE) [3]. G-formula uses Monte Carlo simulations to simulate the outcome, mediators and intermediate confounders under hypothetical interventions or “counter to the fact” scenarios (i.e. contrary to the intervention individuals actually received). The TCE is the value the outcome (e.g. daytime soiling) would take if all individuals had been exposed to a one-unit increase in the FAI versus everyone not being exposed to a one unit increase in the FAI. The NDE is the direct (unmediated) effect of the exposure (e.g. FAI) on the outcome (e.g. daytime soiling) when the mediator(s) (e.g. constipation) takes the value it would be in absence of the exposure (i.e. FAI of 0). More specifically it is modelled as the direct effect of exposure X = x+1 (e.g. an FAI of 1) versus exposure X = x (e.g. an FAI of 0) on outcome Y (e.g. daytime soiling) if mediator M (e.g. constipation) were set to whatever value it would be for X = x. The NIE is the effect of the exposure (e.g. FAI) on the outcome (e.g. daytime soiling) that operates by changing the mediator (e.g. constipation). More specifically, it is modelled as the effect on outcome Y (e.g. daytime soiling) if the exposure X = x+1 and the mediator M (e.g. constipation) were changed from the value it would take if X = x to the value it would take if X = x+1. The proportion of the TCE that is mediated is calculated as $[OR_{NDE} (OR_{NIE} - 1)] / [OR_{NDE} \times OR_{NIE} - 1] \times 100$ [5].

Specifically for this analysis we used a Monte Carlo sample size of 100,000 to minimise fluctuations in effect estimates. Standard errors were estimated using 250 bootstrap samples. We did not hypothesise an interaction between the exposure and mediators and therefore did not include an interaction term within the model. We also specified the command option *linexp* to specify that the exposure is continuous, and its effect is assumed to be linear. For the imputed data we conducted mediation analysis for each of the 50 imputation datasets and output the results to log files. We used R to extract the log ORs and bootstrapped SEs for each of the TCE, NDE, and NIE from each Stata log file. The mean of the log OR was computed and the SEs were calculated using Rubin’s rules [6]. The 95% confidence intervals were calculated from the combined log OR and SEs. Log ORs (and 95% CIs) were exponentiated to represent ORs.

Methods: Missing data

We used multivariate imputation by chained equations under the missing at random assumption to impute missing data [7] in outcome daytime soiling and baseline and

Confidential: For Review Only

intermediate confounders up to the sample with complete data on the exposure FAI score (N = 10,033). We included two types of auxiliary variables; i) additional measures (both before and/or after) of those of the substantive model, and ii) sociodemographic indicators associated with both missingness and many of the variables of the substantive model. These serve two main functions: 1) increasing precision by improving the predictive ability of the imputation model beyond that of the substantive model [8] and 2) making the MAR assumption more plausible by reducing the bias due to data being MNAR [9] (see Supplementary Table S2). All binary variables were imputed using logistic regression, categorical (unordered) variables were imputed using polytomous regression, and all continuous variables were imputed using predictive mean matching. Missing data were imputed separately in males and females before appending the two datasets together. For both males and females 50 imputed datasets with 50 iterations were created using the mice package (version 3.16.0) in R. Estimates were then combined using Rubin's rules [6].

Results: Description of sample

Table 1 in the main manuscript shows the distribution of study variables in the imputed (N = 10 033) and complete case samples (N = 3366). Any daytime soiling was reported for 7.3% of children. In total, 71.7% of children experienced one or more family adversities, the most prevalent being maternal affective disorders (depression, anxiety, and/or suicide attempt; 20.5%). A lack of social support from partner relationships was also common (19.6%; see Supplementary Table 3 for all component prevalences). The mean number of family adversities experienced was 1.9 (Standard Error (SE) 0.02) in all children and was higher in those experiencing soiling (2.3, SE 0.10) compared to those without soiling (1.8, SE 0.02; ttest p-value <0.001). Compared to the imputed data sample, children in the complete case sample experienced fewer adversities and lower proportions of non-white ethnicity, manual social class, preterm birth, parental abuse, and temper tantrums. The complete case sample also had a higher proportion of children with picky eating, hard stools, no maternal smoking during pregnancy, and maternal married status.

Confidential: For Review Only

Supplementary Tables

Table S1 Variable description and coding

Variable name	ALSPAC variable[1]	Time of measure	Measure/Question	Responses	Final variable coding
Exposure					
**Family Adversity Index (FAI)**<br>From 0-2 years	blong	0 to 2 years	DV: long index comprising of 18 items including information on the age of the mother, housing, education, financial status, partner relationship, family, social network, maternal psychopathology, substance abuse and crime	Continuous score (0-18)	Continuous score (0-18)
Outcome					
**Daytime soiling**<br>Frequency child dirties pants during the day	kr125	7.6 years	How often usually does your child dirty his/her pants during the day?	1: Never<br>2: < once /week<br>3: ~ once / week<br>4: 2-5 times / week<br>5: Nearly every day<br>6: > once / day<br>-6; -1: Missing	Recode:<br>1=0: No<br>2/6=1: Yes
Mediators					
**Emotional/behaviour problems**<br>Parent version of the Strengths and Difficulties Questionnaire by Goodman (1997)<br>Total difficulties score	j557f	3.9 years	Derived variable: prorated score of the first 4 subscales (hyperactivity, conduct problems, emotional problems, and peer problems)	Continuous; missing = -1	Continuous (score 0-33)

Confidential: For Review Only

Variable name	ALSPAC variable[1]	Time of measure	Measure/Question	Responses	Final variable coding
**Constipation**	kn1037	5.8 years	In the past 15 months child has had constipation?	1: Yes & Saw Doctor<br>2: Yes But Did Not See Doctor<br>3: Did Not Have<br>-11; -10; -1: Missing	Recode:<br>3=0: No<br>1/2=1: Yes
<u>Baseline Confounders</u>					
**Ethnicity**<br>Child ethnic background	c804	Antenatal	Derived variable: Ethnic group reported for the mother (respondent) or partner:<br>Non-white if c801 or c802 had codes in the range of 2-9	1: White<br>2: Non-white<br>-1: Missing	Recode:<br>1=0: White<br>2=1: Non-white
**Sex**<br>Child's sex assigned at birth	kz021	Birth	Recorded at birth by the fieldworkers who visited the maternity units	1: Male<br>2: Female<br>-2; -1: Missing	Recode:<br>1=0: Male<br>2=1: Female
**Preterm birth**<br>Term vs. preterm	bestgest	Birth	Derived variable: Best guess of gestation when pregnancy ended	Continuous (range 24-47)<br>-11; -10; -3; -2: Missing	Recode:<br>>36 weeks=0: Term<br>≤36 weeks=1: Preterm
**Marital status**	a525	Antenatal	What is your present marital status?	1: Never married<br>2: Widowed<br>3: Divorced<br>4: Separated<br>5: 1st marriage<br>6: Marriage 2 or 3<br>-1: Missing	Recode:<br>5/6=0: Married<br>1=1: Single<br>2/4=2: Divorced/separated/widowed
**Smoking**<br>Maternal smoking during pregnancy	b665 (1st trimester)	Antenatal	Did you smoke regularly in the first three months of pregnancy?	1: No<br>2: Yes, cigarettes<br>3: Yes, cigars<br>5: Yes, other	Recode:<br>[No (0) smoking during trimesters 1, 2 and 3]=0: None<br>[Smoked during 1st trimester (2, 3, 5) but not the 2nd (0) and 3rd (0)]=1: Yes, quit

Confidential: For Review Only

Variable name	ALSPAC variable[1]	Time of measure	Measure/Question	Responses	Final variable coding
	b667 (2nd trimester)	Antenatal	Did you smoke regularly in the last 2 weeks?	1: No<br>2: Yes, cigarettes<br>3: Yes, cigars<br>5: Yes, other	early<br>[Smoked during trimesters 1 (2, 3, 5), 2 (2, 3, 5), and 3 (1, 2, 3) or either 1 and 2 or 1 and 3, or 2 and 3]=2: Yes, throughout
	c483 (3rd trimester)	Antenatal	How many cigarettes per day are you yourself smoking at the moment?	0: None<br>1: 1-9<br>2: 10-19<br>3: 20+<br>-7: Missing	
**Household social class**<br>1991 British Office of Population and Census Statistics (OPCS) job codes (maternal)<br>1991 British Office of Population and Census Statistics (OPCS) job codes (paternal)	c755<br>c765	Antenatal	Derived variable from questions:<br>*Actual job, occupation, trade or profession<br>*Please tick which of the following apply to you: foreman, manager, supervisor, leading hand, self-employed, none of these<br>*Type of industry or service given (main things done in job)	1 - I<br>2 - II<br>3 – III (non-manual)<br>4 – III (manual)<br>5 - IV<br>6 - V<br>65 - Armed forces<br>-1 - missing	Highest parental social class i.e. if one parent is non-manual then social class=0:<br>[Professional, managerial or skilled professions (responses 1, 2, 3)]=0: Non-manual<br>[Partly or unskilled occupations (responses 4, 5, 6)]=1: Manual<br>65=missing
Intermediate Confounders					
**Body mass index (BMI)**	bmi_chdb4		Child health database measures of BMI:<br>Derived from measure of height (m) and weight (kg).<br>Calculated as kg/m2	Continuous measure	Continuous measure
**Difficult temperament**<br>Emotionality, Activity, and Sociability (EAS) Scale	kg620b	3.2 years	Derived variable: EAS emotionality subscale (prorated)	Continuous (score 5-25)<br>-6; -5: Missing	Continuous (score 5-25)

Confidential: For Review Only

Variable name	ALSPAC variable[1]	Time of measure	Measure/Question	Responses	Final variable coding
**Temper tantrums**<br>Frequency child has temper tantrums	kj255	3.5 years	How often does he/she have temper tantrums?	1: Once per Day<br>2: Most Days<br>3: > Once a WK<br>4: < Once a WK<br>5: Never<br>-1: Missing	Recode:<br>3/4/5=0: Few or no temper tantrums<br>1/2=1: Once a day or most days
**Developmental delay**<br>Total developmental score	kf607	2.5 years	Derived variable: prorated scores	Continuous (age-adjusted z-score); missing = -102; -101	Continuous (z score)
**Hard stools**<br>Frequency child's stools are hard	kf141	2.5 years	Nowadays how often are his/her stools hard?	1: Usually<br>2. Sometimes<br>3. Never<br>-1: Missing	Recode:<br>2/3=0: No<br>1=1: Yes
**Stool frequency**<br>Number of motions child has everyday	kf140	2.5 years	Nowadays how many motions (or dirty nappies) a day (24 hours) does he usually have?	0: Other<br>1: 4 or > 4 times<br>2: 2 - 3 times<br>3: once a day<br>4: once in 2 - 4 days<br>5: once a week<br>9: can't say<br>-1: missing	Recode:<br>1/2/3=0: Frequent<br>4/5=1: Infrequent<br>9=missing
**Picky eating**<br>Likes and dislikes of food	kg466	3.2 years	Does your child have definite likes and dislikes as far as food is concerned?	1: No eats most food<br>2. Yes quite choosy<br>3. Yes very choosy<br>-1: Missing	Recode:<br>1/2=0: No<br>3 =1: Yes, picky eater
**Diet: Fibre intake**<br>PC: Healthy eating (proxy for fibre)<br>Northstone et al 2000	kg521	3.2 years	Derived variable: PCA score 2 'healthy' 38 months	Continuous	Continuous
**Maternal bond**<br>Maternal bonding score	h766	2.8 years	Derived variable from h760 + h763	Continuous score (0-33)<br>-1: Missing	Continuous score (0-33)

Confidential: For Review Only

Variable name	ALSPAC variable[1]	Time of measure	Measure/Question	Responses	Final variable coding
**Parental abuse**<br>Parental cruelty (physical and emotional)<br>Question: Have any of these occurred since the study child was 18 months old?	h236a	2.8 years	Partner physically cruel to children	1: Yes<br>2: No<br>-1: Missing	Recode:<br>[h236a, h237a, h247a, h248a: 2]=0: No<br>[any h236a, h237a, h247a, h248a: 1]=1: Yes<br>For imputation derive:<br>Physical cruelty [h236a, h237a: 2]=0: No / [h236a, h237a: 1]=1: Yes<br>Emotional cruelty [h247a, h248a: 2]=0: No / [h247a, h248a: 1]=1: Yes<br>Then passively impute:<br>Physical cruelty = No & Emotional cruelty = No ==0: No parental abuse<br>Physical cruelty = Yes &/or Emotional cruelty = Yes ==1: Yes parental abuse
	h237a	2.8 years	Mother physically cruel to children	1: Yes<br>2: No<br>-1: Missing	
	h247a	2.8 years	Partner emotionally cruel to children	1: Yes<br>2: No<br>-1: Missing	
	h248a	2.8 years	Mother emotionally cruel to children	1: Yes<br>2: No<br>-1: Missing	

[1] ALSPAC variable names; these can be searched for using the ALSPAC variable search tool [http://variables.alspac.bris.ac.uk/].

Confidential: For Review Only

Table S2 Auxiliary variables for multiple imputation of missing data

Daytime soiling in child measured at age 6
Daytime soiling in child measured at age 6
Total difficulties score measured at age 6
Constipation in child measured at age 8
Stool frequency measured at age 3
Hard stools measured at age 3
BMI measured at age 18 months
Picky eating measured at age 2
Healthy eating measured at age 2
House tenure measured in antenatal period
Household crowding measured in antenatal period
Material hardship measured in antenatal period
Antenatal depression in mothers measured in antenatal period
Antenatal anxiety in mothers measured in antenatal period

Confidential: For Review Only

Table S3 Prevalences of the individual adversity components of the family adversity index in (N = 10,033)

FAI constituent	Sub-measure affective group	Prevalence (%)
**Age of mother**	Early parenthood	5.2
**Crime**	Been in trouble with police	3.7
**Crime**	Has convictions	0.6
**Family**	High family size	5.5
**Family**	Has family major problems	0.9
**Financial status**	financial difficulties	15.3
**Housing**	Has housing defects/infestation	17.5
**Housing**	Poor housing adequacy	8.6
**Housing**	Poor housing - basic living	4.8
**Maternal psychopathology**	Has affective disorders (depression, anxiety and/or suicide attempts)	20.5
**No educational qualifications**	Low educational achievement (mother or father)	12.4
**Partner relationship**	Lack of partner social support	19.6
**Partner relationship**	Lack of partner affection	14.7
**Partner relationship**	Experienced partner cruelty	12.9
**Partner relationship**	Partner relationship status	6.5
**Social network**	Lack of emotional support	12.6
**Social network**	Lack of practical/financial support	11.2
**Substance abuse**	Substance/alcohol abuse	13.4

FAI Family adversity index

Confidential: For Review Only

Table S4 Results for the associations between family adversity index and mediators in the imputed data sample (N = 10,033)

Exposure: FAI		Univariable			Multivariable		
		Beta/OR	95% CI	p-value	Beta/OR	95% CI	p-value
Outcome	Emotional/behaviour problems	0.587	0.537, 0.638	<0.001	0.524	0.471, 0.577	<0.001
	Constipation	1.086	1.047, 1.125	<0.001	1.072	1.033, 1.113	<0.001

OR odds ratio; CI confidence interval; FAI family adversity index; all multivariable models adjusted for baseline confounders (sex, ethnicity, preterm birth, household social class, marital status, and smoking during pregnancy). Parameter estimates for emotional/behaviour problems are Betas; constipation are ORs.

Table S5 Results for the associations between emotional/behaviour problems and constipation in the imputed data sample (N = 10,033)

Exposure: Emotional/behaviour problems	Univariable			Multivariable		
	OR	95% CI	p-value	OR	95% CI	p-value
Constipation	1.033	1.017, 1.050	<0.001	1.007	0.986, 1.027	0.521

OR odds ratio; CI confidence interval; multivariable models adjusted for baseline confounders (sex, ethnicity, preterm birth, household social class, marital status, and smoking during pregnancy) and the Family Adversity Index.

Table S6 Results for the associations between family adversity index and mediators with daytime soiling in the complete case sample (N = 3,366)

Outcome: daytime soiling		Univariable			Multivariable		
		OR	95% CI	p-value	OR	95% CI	p-value
Exposure	FAI	1.080	0.998, 1.165	0.051	1.104	1.015, 1.196	0.019
	Emotional/behaviour problems[1]	1.029	0.999, 1.061	0.059	0.995	0.959, 1.032	0.805
	Constipation[1,2]	3.309	2.355, 4.591	<0.001	3.596	2.525, 5.063	<0.001

OR odds ratio; CI confidence interval; FAI family adversity index; all multivariable models adjusted for baseline confounders (sex, ethnicity, preterm birth, household social class, marital status, and smoking during pregnancy). [1] Models additionally adjusted for intermediate confounders (child developmental delay, maternal bonding score, parental abuse, stool frequency, hard stools, difficult temperament, picky eating, healthy eating, temper tantrums, and body mass index), and FAI. [2] Model additionally adjusted for emotional/behaviour problems. Parameter estimates are based on a one-unit increase in FAI, a one-unit increase in emotional/behaviour problems score, and the comparison between yes vs. no for constipation.

Confidential: For Review Only

Table S7 Results for the associations between family adversity index and mediators in the complete case sample (N = 3,366)

Exposure: FAI		Univariable			Multivariable		
		Beta/OR	95% CI	p-value	Beta/OR	95% CI	p-value
Outcome	Emotional/behaviour problems	0.625	0.534, 0.717	<0.001	0.585	0.490, 0.680	<0.001
	Constipation	1.100	1.031, 1.171	0.003	1.086	1.015, 1.160	0.015

OR odds ratio; CI confidence interval; FAI family adversity index; all multivariable models adjusted for baseline confounders (sex, ethnicity, preterm birth, household social class, marital status, and smoking during pregnancy). Parameter estimates for emotional/behaviour problems are Betas; constipation are ORs.

Table S8 Results for the mediation of family adversity index and daytime soiling via emotional/behaviour problems and constipation in the complete case sample (N = 3,366)

Total causal effect		Natural indirect effect		Natural direct effect		PM%
OR	95% CI	OR	95% CI	OR	95% CI	
1.092	1.007, 1.215	0.997	0.957, 1.017	1.096	1.023, 1.017	NA

OR odds ratio; CI confidence interval; PM proportion mediated (%). Mediation models were fitted with emotional/behaviour problems and constipation as the mediators of interest; child developmental delay, maternal bonding score, parental abuse, stool frequency, hard stools, difficult temperament, picky eating, healthy eating, temper tantrums, and body mass index were considered intermediate confounders. All paths were adjusted for baseline confounders: sex, ethnicity, preterm birth, household social class, marital status, and smoking during pregnancy.

Confidential: For Review Only

References

[1] Fraser A, Macdonald-Wallis C, Tilling K, et al. Cohort profile: the Avon Longitudinal Study of Parents and Children: ALSPAC mothers cohort. Int J Epidemiol 2013;42:97–110. https://doi.org/10.1093/ije/dys066.

[2] Boyd A, Golding J, Macleod J, et al. Cohort profile: The 'Children of the 90s'—the index offspring of the Avon Longitudinal Study of Parents and Children. Int J Epidemiol 2013;42:111–27. https://doi.org/10.1093/ije/dys064.

[3] Robins J. A new approach to causal inference in mortality studies with a sustained exposure period—application to control of the healthy worker survivor effect. Mathematical Modelling 1986;7:1393–512. https://doi.org/10.1016/0270-0255(86)90088-6.

[4] De Stavola BL, Daniel RM, Ploubidis GB, et al. Mediation Analysis With Intermediate Confounding: Structural Equation Modeling Viewed Through the Causal Inference Lens. Am J Epidemiol 2015;181:64–80. https://doi.org/10.1093/aje/kwu239.

[5] VanderWeele TJ, Vansteelandt S. Odds Ratios for Mediation Analysis for a Dichotomous Outcome. Am J Epidemiol 2010;172:1339–48. https://doi.org/10.1093/aje/kwq332.

[6] Rubin DB. Multiple Imputation for Nonresponse in Surveys. John Wiley & Sons; 2004.

[7] Royston P, White IR. Multiple Imputation by Chained Equations (MICE): Implementation in Stata. Journal of Statistical Software 2011;45:1–20. https://doi.org/10.18637/jss.v045.i04.

[8] Collins LM, Schafer JL, Kam CM. A comparison of inclusive and restrictive strategies in modern missing data procedures. Psychol Methods 2001;6:330–51.

[9] Cornish RP, Macleod J, Carpenter JR, et al. Multiple imputation using linked proxy outcome data resulted in important bias reduction and efficiency gains: a simulation study. Emerg Themes Epidemiol 2017;14:14. https://doi.org/10.1186/s12982-017-0068-0.

Confidential: For Review Only

Strobe statement

STROBE Statement—checklist of items that should be included in reports of observational studies

	Item No	Recommendation
**Title and abstract**<br>**See title and abstract**	1	(*a*) Indicate the study's design with a commonly used term in the title or the abstract
		(*b*) Provide in the abstract an informative and balanced summary of what was done and what was found
**Introduction**		
Background/rationale<br>**Introduction paragraphs 1 & 2**	2	Explain the scientific background and rationale for the investigation being reported
Objectives<br>**Introduction paragraph 3, Figure 2**	3	State specific objectives, including any prespecified hypotheses
**Methods**		
Study design<br>**Methods**	4	Present key elements of study design early in the paper
Setting<br>**Methods: Sample, Supplementary Text**	5	Describe the setting, locations, and relevant dates, including periods of recruitment, exposure, follow-up, and data collection
Participants<br>**Methods: sample, Figure 1, Supplementary Text**	6	(*a*) *Cohort study*—Give the eligibility criteria, and the sources and methods of selection of participants. Describe methods of follow-up<br>*Case-control study*—Give the eligibility criteria, and the sources and methods of case ascertainment and control selection. Give the rationale for the choice of cases and controls<br>*Cross-sectional study*—Give the eligibility criteria, and the sources and methods of selection of participants
		(*b*) *Cohort study*—For matched studies, give matching criteria and number of exposed and unexposed<br>*Case-control study*—For matched studies, give matching criteria and the number of controls per case
Variables<br>**Methods, Supplementary Tables S1 & S2**	7	Clearly define all outcomes, exposures, predictors, potential confounders, and effect modifiers. Give diagnostic criteria, if applicable
Data sources/ measurement<br>**Methods, Supplementary Tables S1, Supplementary Text**	8*	For each variable of interest, give sources of data and details of methods of assessment (measurement). Describe comparability of assessment methods if there is more than one group
Bias<br>**Methods: Statistical analysis and missing data, Supplementary Text, Discussion**	9	Describe any efforts to address potential sources of bias
Study size	10	Explain how the study size was arrived at

Confidential: For Review Only

**Methods: Sample, Figure 1, Supplementary Text**		
Quantitative variables **Methods: Statistical analysis, Supplementary Table S1**	11	Explain how quantitative variables were handled in the analyses. If applicable, describe which groupings were chosen and why
Statistical methods **Methods: Statistical analysis, Supplementary Text**	12	(*a*) Describe all statistical methods, including those used to control for confounding
		(*b*) Describe any methods used to examine subgroups and interactions
		(*c*) Explain how missing data were addressed
		(*d*) *Cohort study*—If applicable, explain how loss to follow-up was addressed<br>*Case-control study*—If applicable, explain how matching of cases and controls was addressed<br>*Cross-sectional study*—If applicable, describe analytical methods taking account of sampling strategy
		(*e*) Describe any sensitivity analyses
**Results**		
Participants **Figure 1, Supplementary Text, Table 1**	13*	(a) Report numbers of individuals at each stage of study—eg numbers potentially eligible, examined for eligibility, confirmed eligible, included in the study, completing follow-up, and analysed
		(b) Give reasons for non-participation at each stage
		(c) Consider use of a flow diagram
Descriptive data **Table 1, Results**	14*	(a) Give characteristics of study participants (eg demographic, clinical, social) and information on exposures and potential confounders
		(b) Indicate number of participants with missing data for each variable of interest
		(c) *Cohort study*—Summarise follow-up time (eg, average and total amount)
Outcome data **Table 1, Results**	15*	*Cohort study*—Report numbers of outcome events or summary measures over time
		Case-control study—Report numbers in each exposure category, or summary measures of exposure
		Cross-sectional study—Report numbers of outcome events or summary measures
Main results **Results, Tables 1-3, Supplementary Tables S3-7**	16	(*a*) Give unadjusted estimates and, if applicable, confounder-adjusted estimates and their precision (eg, 95% confidence interval). Make clear which confounders were adjusted for and why they were included
		(*b*) Report category boundaries when continuous variables were categorized
		(*c*) If relevant, consider translating estimates of relative risk into absolute risk for a meaningful time period
Other analyses **Complete case analysis Supplementary Tables S5-7**	17	Report other analyses done—eg analyses of subgroups and interactions, and sensitivity analyses
**Discussion**		

Confidential: For Review Only

Key results **Discussion paragraph 1**	18	Summarise key results with reference to study objectives
Limitations **Discussion: Strengths and limitations**	19	Discuss limitations of the study, taking into account sources of potential bias or imprecision. Discuss both direction and magnitude of any potential bias
Interpretation **Discussion: Comparison with previous findings and potential mechanisms**	20	Give a cautious overall interpretation of results considering objectives, limitations, multiplicity of analyses, results from similar studies, and other relevant evidence
Generalisability **Discussion: Strengths and limitations**	21	Discuss the generalisability (external validity) of the study results
**Other information**		
Funding **Funding section**	22	Give the source of funding and the role of the funders for the present study and, if applicable, for the original study on which the present article is based

*Give information separately for cases and controls in case-control studies and, if applicable, for exposed and unexposed groups in cohort and cross-sectional studies.

Note: An Explanation and Elaboration article discusses each checklist item and gives methodological background and published examples of transparent reporting. The STROBE checklist is best used in conjunction with this article (freely available on the Web sites of PLoS Medicine at http://www.plosmedicine.org/, Annals of Internal Medicine at http://www.annals.org/, and Epidemiology at http://www.epidem.com/). Information on the STROBE Initiative is available at www.strobe-statement.org.

Confidential: For Review Only

BMJ Paediatrics Open

Examining causal pathways between family adversity and soiling: a prospective cohort study

Journal:	*BMJ Paediatrics Open*
Manuscript ID	bmjpo-2025-003978.R1
Article Type:	Original research
Date Submitted by the Author:	26-Nov-2025
Complete List of Authors:	Burrows, Kimberley; University of Bristol Medical School, Centre for Academic Child Health, Population Health Sciences<br>Heron, Jon; University of Bristol Medical School, Centre for Academic Mental Health, Population Health Sciences; University of Bristol Medical School, Medical Research Council Integrative Epidemiology Unit, Population Health Sciences<br>Richardson, Davina; Bladder and Bowel UK, The Toilet Training Team<br>Rayner, Juliette; ERIC, The Children's Bowel and Bladder Charity<br>Spray, Chris; Bristol Royal Hospital for Children, Paediatric Gastroenterology Department<br>Joinson, Carol; University of Bristol Medical School, Centre for Academic Child Health, Population Health Sciences
Keywords:	Child Health, Epidemiology, Statistics, Enuresis

SCHOLARONE™
Manuscripts

I, the Submitting Author has the right to grant and does grant on behalf of all authors of the Work (as defined in the below author licence), an exclusive licence and/or a non-exclusive licence for contributions from authors who are: i) UK Crown employees; ii) where BMJ has agreed a CC-BY licence shall apply, and/or iii) in accordance with the terms applicable for US Federal Government officers or employees acting as part of their official duties; on a worldwide, perpetual, irrevocable, royalty-free basis to BMJ Publishing Group Ltd ("BMJ") its licensees and where the relevant Journal is co-owned by BMJ to the co-owners of the Journal, to publish the Work in this journal and any other BMJ products and to exploit all rights, as set out in our licence.

The Submitting Author accepts and understands that any supply made under these terms is made by BMJ to the Submitting Author unless you are acting as an employee on behalf of your employer or a postgraduate student of an affiliated institution which is paying any applicable article publishing charge ("APC") for Open Access articles. Where the Submitting Author wishes to make the Work available on an Open Access basis (and intends to pay the relevant APC), the terms of reuse of such Open Access shall be governed by a Creative Commons licence – details of these licences and which Creative Commons licence will apply to this Work are set out in our licence referred to above.

Other than as permitted in any relevant BMJ Author's Self Archiving Policies, I confirm this Work has not been accepted for publication elsewhere, is not being considered for publication elsewhere and does not duplicate material already published. I confirm all authors consent to publication of this Work and authorise the granting of this licence.

Confidential: For Review Only

TITLE

Examining causal pathways between family adversity and soiling: a prospective cohort study

AUTHORS

Kimberley Burrows PhD[1], Jon Heron PhD[2,3], Davina Richardson[4], Christine Spray[5], Juliette Rayner[6], Carol Joinson PhD [1]

INSTITUTIONS

1. Centre for Academic Child Health, Population Health Sciences, Bristol Medical School, at the University of Bristol, Bristol, United Kingdom.
2. Centre for Academic Mental Health, Population Health Sciences, Bristol Medical School, at the University of Bristol, Bristol, United Kingdom.
3. Medical Research Council Integrative Epidemiology Unit, Population Health Sciences, Bristol Medical School, at the University of Bristol, Bristol, United Kingdom.
4. Children's Nurse Specialist, Bladder and Bowel UK, The Toilet Training Team.
5. Paediatric Gastroenterology Department, Bristol Royal Hospital for Children, Bristol BS2 8BJ, United Kingdom.
6. ERIC, The Children's Bowel and Bladder Charity, 36 Old School House, Kingswood Foundation, Brittania Rd, Bristol, BS15 8DB, United Kingdom.

Corresponding author: Kimberley Burrows, Centre for Academic Child Health, Population Health Sciences, Bristol Medical School, University of Bristol, Canynge Hall, 39 Whatley Road, Bristol BS8 2PS. Email address: Kimberley.Burrows@bristol.ac.uk

Confidential: For Review Only

Key words: ALSPAC; Cohort Studies; Mediation Analysis; gformula, Constipation, Faecal Incontinence, Adversity

Word count: 2990

Confidential: For Review Only

Abstract [249/300]

Objective: We aimed to examine the associations between family adversity in infancy with subsequent daytime soiling at primary school age. We also examined factors on the casual pathway between family adversity and soiling, specifically the child's emotional/behaviour problems and constipation.

Design: This prospective cohort study included children from the Avon Longitudinal Study of Parents and Children (*N*=10 033) with data on the Family Adversity Index (FAI; birth to age 2), daytime soiling at age 7.5, emotional/behaviour problems at age 4, and constipation at age 6. We examined the relationships between the FAI, soiling, emotional/behaviour problems, and constipation. We then evaluated the mediating effects of emotional/behaviour problems and constipation. All analyses were adjusted for child and family related confounders.

Results: Daytime soiling occurred in 7% of children. A one-unit increase in infancy FAI was associated with increased odds of 12% for daytime soiling at age 7.5 (odds ratio [OR] 1.12, 95% confidence interval [CI] 1.07, 1.17), emotional/behaviour problems score at age 4 (beta 0.52, 95%CI 0.47, 0.58) and increased odds of constipation at age 6 (OR 1.07, 95%CI 1.03, 1.11). The presence of constipation at age 6 was strongly associated with increased odds of soiling at age 7.5 (OR 3.34, 95%CI 2.68, 4.16). There was weak evidence that the associations between FAI and daytime soiling were mediated by emotional/behaviour problems and constipation ($OR_{natural\_indirect\_effect}$ 1.03, 95%CI 0.99-1.07).

Conclusions: Clinicians should be aware that exposure to family adversity in the early years places children at greater risk of subsequent constipation and soiling.

Key messages

What is already known on this topic?

- There is growing evidence that adversities including exposure to stressful life events, maternal psychopathology, and family discord may play a role in determining a child's later risk of soiling.

What this study adds?

- This is the first study to examine mechanisms explaining the relationship between adversity and soiling using causal mediation methods in a large community-based birth cohort study.
- The total burden of family adversity (encompassing facets of psychopathology, discord and events) during infancy increases the odds of soiling at primary school age.
- There is weak evidence that emotional/behaviour problems and constipation mediate the relationship between family adversity and soiling.

How this study might affect research, practice or policy?

- Clinicians should be aware that exposure to family adversity in the early years places children at greater risk of subsequent soiling.
- Increased resources need to be directed at providing support and interventions for families who are experiencing adversities to reduce the impact on children's health and development.

Confidential: For Review Only

INTRODUCTION

Soiling (faecal incontinence) is a common paediatric health problem (estimated prevalence of 4% in primary school-age children) [1], which causes significant distress to children and their parents [2] as well as presenting challenges within educational settings. Most cases of childhood soiling are functional and are a consequence of overflow due to chronic constipation [3]. Stool withholding and toileting refusal, due to early painful experiences of passing hard stools, are primary causes of functional constipation in children [3].

There is growing evidence that adversity in the child's early family environment plays a role in determining a child's later risk of soiling. Previous studies have focused on specific types of adversities including exposure to stressful life events, maternal psychopathology, and family discord [4–8], but none have examined the total burden of exposure to a range of adversities in the early years and many were cross-sectional or used a retrospective design (reviewed in [4,8]). There is also a lack of research examining mechanisms that could explain how early adverse exposures could increase a child's subsequent risk of soiling.

The current paper, based on data from a large UK birth cohort, examines the prospective relationship between exposure to family adversity in infancy (0-2 years) and subsequent soiling at primary school age (7.5 years). We use a measure of family adversity which comprises multiple domains including deprivation, low parental education, parental discord, parental crime, parental psychopathology, and parents' lack of social/practical/emotional support. We also examine factors on the causal pathway between family adversity and soiling, specifically the child's emotional/behaviour problems and constipation. We chose to focus on these factors

Confidential: For Review Only

because these are potentially modifiable and could be targeted in interventions aimed at reducing the risk of soiling [6,9–13].

METHODS

We used the Strengthening the Reporting of Observational Studies in Epidemiology (STROBE) cohort reporting guidelines (see STROBE checklist in the Supplementary Text).

Sample

ALSPAC is a prospective, population-based birth cohort study that recruited pregnant women resident in Avon, UK with expected dates of delivery between 1st April 1991 and 31st December 1992 [14,15]. The initial number of pregnancies enrolled was 14 541 resulting in 14 062 live births and 13 988 children who were alive at 1 year of age. Further details are given in the Supplementary Text.

The eligible sample for this study included children who had available data on the family adversity index score (FAI; see below) derived for the period between birth and two years of age (N = 10 033). Complete data for the FAI, mediators, daytime soiling, and confounders were available for 3366 children. The participant flowchart is presented in Figure 1.

Patient and public involvement

Patients and/or the public were not involved in the design, conduct, reporting or dissemination of this research.

Exposure: family adversity index

Confidential: For Review Only

Multiple family, parental, sociodemographic, and social support indicators were assessed via questionnaires completed during pregnancy and when the child was 0-2 years old. The FAI consisted of 18 items about the age of the mother at pregnancy, housing, parents' education, financial difficulties, relationship status and support, family size and care, social network, substance abuse, crime, and psychopathology of parents. Total FAI score was derived by summing all 18 items, with higher scores indicating greater family adversity. See Supplementary Text and Supplementary Table 1 for further information on the FAI.

Outcomes: daytime soiling

When the study children were 7 years 8 months (SD = 0.14) (hereafter referred to as 7.5 years) mothers were asked "how often does your child dirty their pants during the day". Options included "Never"; "Occasional accidents but less than once a week"; "About once a week"; "2-5 times a week"; "Nearly every day"; and "More than once a day". After exploring the proportions of respondents for the more frequent soiling categories (only 0.83% responded with 2-5 times a week or more), we chose to derive a binary variable to indicate any daytime soiling by assigning a value of 0 to "Never" and 1 to all other categories. Further information for coding daytime soiling is given in Supplementary Table 1.

Mediators: emotional/behaviour problems and constipation

We chose emotional/behavioural problems and constipation as mediators as these are potentially modifiable and could be targeted in interventions aimed at reducing the risk of soiling [6,9–13]. Furthermore, there is evidence that emotional/behavioural problems are associated with constipation and soiling [16,17] and so we considered the two mediators jointly within our causal mediation models (see Figure 2).

Confidential: For Review Only

When the study child was 4 years (SD = 0.12), mothers completed the Strengths & Difficulties Questionnaire (SDQ) [18], which comprises five subscales (each with five items: hyperactivity, conduct problems, emotional symptoms, peer problems and prosocial behaviour). An emotional/behaviour problems score was calculated from the SDQ using the first four subscales (range: 0 to 40). A high score indicates more emotional/behaviour problems.

When the study child was 5 years 10 months (SD = 0.12) (hereafter referred to as 6 years) mothers were asked “Has your child had any constipation in the past 12 months”. Options included “Yes and saw a doctor”; “Yes but did not see a doctor”; “No, did not have”. We derived a binary variable to indicate constipation by assigning a value of 0 to “No, did not have” and 1 to the other two other categories. Further information for coding the mediators is in Supplementary Table 1.

Baseline and intermediate confounders

We adjusted for baseline confounders (measured during the antenatal period or at birth) of each of the mediating pathways (exposure-outcome, exposure-mediator, and mediator-outcome). Confounders measured during the antenatal period included ethnicity, household social class, marital status, and smoking during pregnancy. The confounders sex assigned at birth and preterm birth were measured during the postnatal period. Other indicators of socioeconomic status and maternal psychopathology were intrinsically linked to the factors included in the FAI (either directly or measured very closely in time to those included in the FAI) and were omitted as confounders.

We also included intermediate confounders in our models i.e. factors that may be on the causal pathway from the exposure (FAI) to the mediators (emotional/behaviour problems and constipation) (see Figure 2) and thus confound the mediate-outcome

Confidential: For Review Only

relationships. These included child developmental delay, maternal bonding score, parental abuse, stool frequency, hard stools, difficult temperament, picky eating, healthy eating, temper tantrums, and BMI all measured between ages 30 to 45 months [6,9–13]. Figure 2Details on the measurement and coding of the confounders and intermediate confounders are given in Supplementary Table 1.

Statistical analysis

We used univariable and multivariable logistic and linear regression to examine the relationships between (i) FAI and daytime soiling, (ii) mediators and daytime soiling, and (iii) FAI and mediators (emotional/behaviour problems and constipation).

We used the *gformula* (parametric g-computation formula [19]) command in Stata [20] to examine the mediating effect of emotional/behaviour problems and constipation jointly on the association between FAI score and daytime soiling (see Figure 2). G-formula is based on the counterfactual framework and allows the decomposition of effects into the total causal effect (TCE), the natural indirect effect (NIE) (via the mediators of interest), and the natural direct effect (NDE) (not via the mediators of interest). G-formula can also estimate marginal odds ratios for mediation effects with a binary outcome whilst incorporating intermediate confounders (see Supplementary Text for further information). We used g-formula to decompose the NIE via emotional/behaviour problems and constipation jointly as we did not consider them to be independent. We used a Monte Carlo sample size of 100 000 and we estimated normal-based 95% confidence intervals (CI) using standard errors from 250 bootstrap resamples. The proportion mediated (PM) was calculated as $[OR_{NDE} (OR_{NIE} - 1)] / [OR_{NDE} \times OR_{NIE} - 1] \times 100$ however, this is only interpretable when the TCE, NIE, and NDE have the same direction of effect [21].

Analyses were conducted using Stata version 18 [22] and R version 4.3.0 [23].

Confidential: For Review Only

Missing data

We used multivariate imputation by chained equations under the missing at random assumption to impute missing data [24] in daytime soiling and baseline and intermediate confounders up to the sample with complete data on the FAI score (N = 10 033). We also included auxiliary variables that are considered predictive of the missing values including socioeconomic status indicators and earlier measures of variables (see Supplementary Table 2). Missing data were imputed separately in males and females before appending the two datasets together. For both males and females 50 imputed datasets with 50 iterations were created using the mice package (version 3.16.0) in R. Estimates were then combined using Rubin's rules [25] (see Supplementary Text).

RESULTS

Table 1 shows the distribution of study variables in the imputed (N = 10 033) and complete case samples (N = 3366). Any daytime soiling was reported for 7.3% of children. In total, 71.7% of children experienced one or more family adversities with the mean number of family adversities experienced was 1.9 (Standard Error (SE) 0.02) in all children and was higher in those experiencing soiling (2.3, SE 0.10) compared to those without soiling (1.8, SE 0.02; ttest p-value <0.001). Further details of the study sample and variables are given in the Supplementary text (Results: description of sample) and Supplementary Table 3.

Confidential: For Review Only

Table 1 Descriptive statistics of study variables for the imputed sample (N = 10 033) and complete case sample (N = 3366)

Variable	Eligible sample (N =< 10,033)		N Missing from eligible sample (N = 10,033)[1]	Imputed sample (N = 10,033)		Complete case sample (N = 3,366)	
	Mean	SE	N [%]	Mean	SE	Mean	SE
FAI (0-13)[2]	1.86	0.02	NA	1.86	0.02	1.56	0.03
Child development level (Z-score)	0.01	0.01	2285 [22.8]	0.01	0.01	-0.01	0.02
Maternal bonding score (10-33)	27.63	0.04	1701 [17.0]	27.61	0.04	27.72	0.06
Child difficult temperament (5-25)	12.43	0.05	1307 [13.0]	12.45	0.05	12.34	0.07
Healthy diet (principal component score)	-0.01	0.01	1345 [13.4]	-0.01	0.01	-0.04	0.02
BMI (kg/m2)	16.23	0.02	3131 [31.2]	16.23	0.02	16.22	0.02
Emotional/behaviour problems score (0-40)	8.77	0.05	1791 [17.9]	8.85	0.05	8.53	0.08
	%	SE	N [%]	%	SE	%	SE
Childs sex assigned at birth (female)	48.73	0.5	NA	48.73	0.5	49.67	0.86
Maternal smoking during pregnancy							
None	79.06	0.41	121 [1.2]	78.95	0.41	84.46	0.62
Yes, quit early	5.14	0.22		5.16	0.22	3.81	0.34
Yes, throughout	15.81	0.37		15.89	0.37	11.74	0.55
Marital status							
Married	81.42	0.39	155 [1.5]	81.28	0.39	86.69	0.59
Single	13.74	0.35		13.84	0.35	9.63	0.51
Divorced/separated/widowed	4.84	0.22		4.88	0.22	3.68	0.32
Household social class (manual)	16.67	0.38	519 [5.17]	17.27	0.39	12.33	0.57
Preterm birth	4.73	0.21	NA	4.73	0.21	4.43	0.35
Ethnicity (non-white)	3.82	0.19	154 [1.5]	3.88	0.2	2.47	0.27
Stool frequency (low)	4.97	0.23	1368 [13.6]	5.14	0.24	5.53	0.39
Hard stools	27.93	0.48	1214 [12.1]	28.08	0.48	29.11	0.78
Parental abuse	4.57	0.23	1589 [15.8]	5.09	0.26	4.04	0.34
Picky eating	14.79	0.38	1298 [12.9]	14.83	0.37	15.06	0.62

Temper tantrums	15.55	0.39	1399 [13.9]	15.98	0.4	14.05	0.6
Constipation at 6 years	10.27	0.35	2488 [24.8]	10.77	0.37	10.49	0.53
Daytime soiling	6.72	0.29	2806 [28.0]	7.3	0.3	7	0.44

FAI family adversity index; BMI body mass index. 50 imputed datasets. The complete case sample comprises individuals with data for all variables described. [1] The total number of missing participants for each variable. NA none missing. [2] The FAI is not normally distributed, the median (IQR) is 1 (0-3).

Table 2 presents strong evidence that a one-unit increase in the FAI score was associated with increased odds of 12% for daytime soiling at age 7.5 (adjusted Odds Ratio (OR) 1.12, 95% confidence interval (CI) 1.07, 1.17) after adjustment for confounders. We also found that a one-unit increase in the emotional/behaviour problems score at age 4 was associated with an increase in the odds of daytime soiling at age 7.5 (adjusted OR 1.03, 95% CI 1.002, 1.05). The presence of constipation at age 6 was strongly associated with increased odds of soiling at age 7.5 (adjusted OR 3.34, 95% CI 2.68, 4.16).

Table 2 Results for the associations between family adversity index and mediators with daytime soiling in the imputed data sample (N = 10 033)

Outcome: daytime soiling		Univariable			Multivariable		
		OR	95% CI	p-value	OR	95% CI	p-value
Exposure	FAI	1.11	1.06, 1.16	<0.001	1.12	1.07, 1.17	<0.001
	Emotional/behaviour problems[1]	1.07	1.05, 1.09	<0.001	1.03	1.00, 1.05	0.04
	Constipation[1,2]	3.32	2.69, 4.10	<0.001	3.34	2.68, 4.16	<0.001

OR odds ratio; CI confidence interval; FAI family adversity index; all multivariable models adjusted for baseline confounders (sex, ethnicity, preterm birth, household social class, marital status, and smoking during pregnancy). [1] Models additionally adjusted for intermediate confounders (child developmental delay, maternal bonding score, parental abuse, stool frequency, hard stools, difficult temperament, picky eating, healthy eating, temper tantrums, and BMI), and FAI. [2] Model additionally adjusted for emotional/behaviour problems. Analysis performed in 50 imputed datasets. Parameter estimates are based on a one-unit increase in FAI, a one-unit increase in emotional/behaviour problems score, and the comparison between yes vs. no for constipation.

A one-unit increase in the FAI score was associated with an increase in the emotional/behaviour problems score (adjusted beta 0.52, 95% CI 0.47, 0.58) and

Confidential: For Review Only

increased odds of constipation (adjusted OR 1.07, 95% CI 1.03, 1.11**;** Supplementary Table 4). There was weak evidence that a one-unit increase in the emotional/behaviour problems score was associated with constipation (adjusted OR 1.01, 95% CI 0.99, 1.03**;** Supplementary Table 5).

Table 3 shows the TCE, NIE, NDE, and the proportion mediated of the one-unit increase in FAI on daytime soiling jointly through the mediators emotional/behaviour problems and constipation. There was weak evidence of a mediating effect (OR_{NIE} 1.03, 95% CI 0.99, 1.07, PM 27**%**) however, the effects are imprecise with the 95% CI crossing the null.

As a sensitivity analysis, we repeated all analyses with the complete-case sample (N = 3366**;** Supplementary Tables 6-8**)**. The direction and magnitude of effects were consistent with the imputed data results.

Table 3 Results for the mediation of family adversity index and daytime soiling via emotional/behaviour problems and constipation in the imputed data sample (N = 10 033)

**Outcome**	**Total causal effect**		**Natural indirect effect**		**Natural direct effect**		**PM%**
	**OR**	**95% CI**	**OR**	**95% CI**	**OR**	**95% CI**	
Daytime soiling	1.10	1.04, 1.17	1.03	0.99, 1.07	1.07	1.01, 1.14	27

OR odds ratio; CI confidence interval; PM proportion mediated (%). Mediation models were fitted with emotional/behaviour problems and constipation as the mediators of interest; child developmental delay, maternal bonding score, parental abuse, stool frequency, hard stools, difficult temperament, picky eating, healthy eating, temper tantrums, and BMI were considered intermediate confounders. All paths were adjusted for baseline confounders: sex, ethnicity, preterm birth, household social class, marital status, and smoking during pregnancy. Analysis performed in 50 imputed datasets.

DISCUSSION

This is the first study to examine mechanisms that could explain the relationship between early childhood adversity and subsequent soiling at primary school age. Using data from a large community-based birth cohort study with a wide range of

Confidential: For Review Only

confounders, we found that children who were exposed to family adversity in the first two years of life were at greater risk of soiling at 7.5 years. There was weak evidence that emotional/behaviour problems and constipation mediated this relationship. Notably, we found that early exposure to family adversity increased the risk of subsequent constipation at age 6. Emotional/behaviour problems at age 4 were weakly associated with constipation at age 6. Consistent with previous studies, we found that the presence of constipation was strongly associated with an increased risk of subsequent soiling.

Comparison with previous findings and potential mechanisms

We found strong evidence that early exposure to family adversity increased the risk of subsequent soiling and constipation. Family-related stressors have previously been linked to constipation in cross-sectional studies [5,26]. There is evidence of the effect of stress on bowel functioning through the gut-brain axis; a bidirectional communication system including the neural, endocrine, microbial, and immune system that connects the two [27]. For example, modulation of bowel motility, secretion, and mucosal responses are controlled by the brain [28]. Development of the gut-brain axis during early infancy and childhood may be impacted by psychosocial factors including stressful events and adversity [27] and may be a plausible mechanism through which early family adversity increases risk of later constipation and soiling.

We found only weak evidence for the association between emotional/behavioural problems score at age 4 and constipation at age 6. This is contrary to a previous study using ALSPAC data, which reported strong evidence of associations between emotional/behavioural problems at 3.5 years and trajectories of constipation and soiling from age 4-10 years [6]. However, the current study uses a measure of

Confidential: For Review Only

constipation at a single timepoint, compared with the previous study which examined trajectories comprising repeated measures of soiling and constipation across childhood.

We found only weak evidence that emotional/behaviour problems and constipation mediated the relationship between family adversity and children's soiling. It is possible that family adversity is linked to an increased risk of childhood soiling through other causal pathways. For example, inadequate toilet training practices have been linked to an increased risk of bowel disorders [29]. ALSPAC did not collect data on the strategies employed by parents during toilet training but did collect data on age at initiation of toilet training. However, there was little evidence that age at initiation of toilet training was associated with constipation and/or soiling at primary school age [11]. Further research is needed to examine toilet training practices as possible mediators of the link between family adversity and soiling.

Data were not available on important factors at preschool age that are associated with the development of childhood constipation including stool withholding and toileting refusal. However, our analysis did include hard stools and low stool frequency at age 2½ years as intermediate confounders. These problems can be both causes and consequences of stool withholding and toileting refusal. It has previously been argued that stool withholding is unlikely to be caused primarily by emotional/behaviour problems, since painful defecation usually precedes the onset of stool toileting refusal [30]. However, earlier studies based on small clinic samples have found evidence for more difficult temperament traits in children who are difficult to toilet train, and in children with stool toileting refusal [31,32]. There is also evidence that autistic traits and ADHD are associated with soiling, and it is suggested that this could be due to sensory issues which lead to stool withholding and constipation [33,34].

Confidential: For Review Only

At primary school-age, the focus of this study, other causes of stool withholding include limited access to toilets, hygiene concerns, and perceived lack of privacy or safety of school toilets [35]. Further research is needed to determine the causes of stool toileting refusal in children at different ages and to advance understanding of factors that cause lasting vulnerability to soiling. Identification of biopsychosocial factors on the causal pathway from adversity to soiling could lead to the development of novel therapeutic targets.

Strengths and limitations

Major strengths of this study include the use of data from a large birth cohort, the prospective design, the availability of data on a wide range of relevant confounders and intermediate confounders, and the use of causal mediation methods to examine causal pathways between family adversity in early childhood and subsequent risk of soiling at school age.

We did not restrict our analysis to children who met clinical diagnostic criteria for faecal incontinence, therefore, the study findings apply to children with soiling in the community and not just those with the most severe soiling [11]. This is a community-based sample, and therefore, the proportion of participants who experienced soiling at high frequencies is small compared with clinical samples (0.83%). We therefore examined the presence versus absence of any soiling and did not further categorise by frequency because this would have resulted in very small group sizes and lack of precision in our estimates. It is important to note that we found robust associations between family adversity and soiling, even when examining soiling that did not meet the criteria for clinical diagnosis [36]. We also acknowledge that any level of soiling, even if infrequent is distressing for children at primary school age and can lead to peer

Confidential: For Review Only

rejection and social isolation [37]. Parents were not asked about the duration of constipation or soiling at each time point, but the repeated measures of constipation and soiling in the ALSPAC cohort indicate that constipation and soiling are chronic problems [11].

The complete-case sample had a lower proportion of participants with socioeconomic disadvantage (e.g. manual social class and divorced/separated/widowed marital status) and FAI than the wider ALSPAC cohort, which may limit the internal validity of our findings. We therefore used multiple imputation to address possible bias due to missing data and report all results as recommended by Sterne et al. [38].

Reliance on parental reports of constipation is a possible limitation. Parents were asked to report if their child had suffered from constipation in the past 12 months and if they had seen doctor but were not asked about the frequency or severity of constipation. Seeing a doctor for constipation is not necessarily an indicator of severity but might be determined by factors such as parental health literacy. Underreporting of constipation is possible since some parents may have been unaware that their child is constipated. However, the fact that the prevalence of constipation in ALSPAC is similar to other population-based studies [39] provides reassurance concerning the validity of the parental reports. The use of parental reports of soiling and the specific phrasing of the question ("how often does your child dirty their pants during the day") could have led to the possibility of parents responding 'yes' to the soiling question due to their child's inadequate wiping following a bowel movement (rather than an episode of faecal incontinence). It is notable, however, that some parents stated in the questionnaire that their child had soiled his/her pants only because of poor wiping or due to suffering an episode of diarrhoea and in these cases they did not place their child in the soiling category [6].

Confidential: For Review Only

We did not differentiate between retentive and non-retentive subtypes of soiling. Clinical studies, however, estimate that over 80% of children who experience functional faecal incontinence have underlying constipation [36]. We also did not examine nocturnal soiling as an outcome at age 7.5, but the prevalence was very low at this age in the ALSPAC cohort (0.8%).

Implications

Clinicians should be aware that exposure to family adversity in the early years places children at greater risk of subsequent constipation and soiling. Screening for adversities could be conducted at routine contacts with health visitors, GPs, and in paediatric bladder and bowel clinics. Increased resources need to be directed at providing support and interventions for families who are experiencing adversities to reduce the impact on children's health and development.

Constipation in preschool children is still not universally understood and managed by primary care practitioners in much of the UK, despite NICE guidance and a wide evidence base for proactive treatment [40]. There are still some areas in the UK without bladder and bowel services for children and other areas where services do exist but are restricted to seeing children aged over 5 years or are inadequately resourced resulting in long wait times for children to receive specialist assessment and support. This is a serious concern because chronic constipation and soiling in children have adverse impacts on their quality of life and psychosocial functioning [40]. Our findings confirm the importance of training for all health care professionals, early years practitioners and parents/carers on signs and symptoms of constipation in children from birth onwards to enable prompt intervention before it becomes chronic.

Confidential: For Review Only

CONTRIBUTORS

CJ, JH, KB: conceptualisation; CJ, JH: funding acquisition; KB, CJ, JH: analytical plan; KB, JH, CJ: methodology; KB: formal analysis; KB, CJ, JH, DR, JR, CS: interpretation of findings; KB, CJ: writing original draft; KB, CJ, JH, DR, JR, CS: writing review and editing. This publication is the work of the authors and Kimberley Burrows will serve as guarantors for the contents of this paper.

COMPETING INTERESTS

None declared

FUNDING

This work is supported by funding from the Medical Research Council [grant ref: MR/V033581/1: Mental Health and Incontinence]. JH is a member of the MRC Integrative Epidemiology Unit at the University of Bristol [MC_UU_00011/7]. The UK Medical Research Council and Wellcome [grant ref: 217065/Z/19/Z] and the University of Bristol provide core support for ALSPAC. This publication is the work of the authors and Kimberley Burrows will serve as guarantors for the contents of this paper. A comprehensive list of grants funding is available on the ALSPAC website: http://www.bristol.ac.uk/alspac/external/documents/grant-acknowledgements.pdf.

ACKNOWLEDGEMENTS

We are extremely grateful to all the families who took part in this study, the midwives for their help in recruiting them, and the whole ALSPAC team, which includes interviewers, computer and laboratory technicians, clerical workers, research scientists, volunteers, managers, receptionists, and nurses.

DATA AVAILABILITY

Confidential: For Review Only

The ALSPAC data underlying this article are available upon request from the ALSPAC Executive Committee for researchers who meet the criteria for access to confidential data (https://bristol.ac.uk/alspac/researchers/access/). Code to conduct these analyses can be found on https://github.com/burrowsk/Family-adversity-constipation-soiling.

Ethics approval

Ethical approval for the study was obtained from the ALSPAC Ethics and Law Committee and the Local Research Ethics Committees. Research ethics committee approval references can be found at http://www.bristol.ac.uk/alspac/researchers/research-ethics/. Initial approval references were granted by Bristol and Weston Health Authority (E1808), Southmead Health Authority (49/89), and Frenchay Health Authority (90/8). Informed consent for the use of all data collected was obtained from participants following the recommendations of the ALSPAC Ethics and Law Committee at the time. Participants can contact the study team at any time to retrospectively withdraw consent for their data to be used. Study participation is voluntary and during all data collection sweeps, information was provided on the intended use of data.

SUPPLEMENTAL MATERIAL

This content has been supplied by the author(s). It has not been vetted by BMJ Publishing Group Limited (BMJ) and may not have been peer-reviewed. Any opinions or recommendations discussed are solely those of the author(s) and are not endorsed by BMJ. BMJ disclaims all liability and responsibility arising from any reliance placed on the content. Where the content includes any translated material, BMJ does not

Confidential: For Review Only

warrant the accuracy and reliability of the translations (including but not limited to local regulations, clinical guidelines, terminology, drug names and drug dosages), and is not responsible for any error and/or omissions arising from translation and adaptation or otherwise.

Confidential: For Review Only

REFERENCES

[1] van der Wal MF, Benninga MA, Hirasing RA. The prevalence of encopresis in a multicultural population. J Pediatr Gastroenterol Nutr 2005;40:345–8. https://doi.org/10.1097/01.mpg.0000149964.77418.27.

[2] Kovacic K, Sood MR, Mugie S, et al. A multicenter study on childhood constipation and fecal incontinence: effects on quality of life. J Pediatr 2015;166:1482-1487.e1. https://doi.org/10.1016/j.jpeds.2015.03.016.

[3] Koppen IJN, Benninga MA. Functional Constipation and Dyssynergic Defecation in Children. Front Pediatr 2022;10:832877. https://doi.org/10.3389/fped.2022.832877.

[4] Philips EM, Peeters B, Teeuw AH, et al. Stressful Life Events in Children With Functional Defecation Disorders. J Pediatr Gastroenterol Nutr 2015;61:384–92. https://doi.org/10.1097/MPG.0000000000000882.

[5] Oswari H, Alatas FS, Hegar B, et al. Epidemiology of Paediatric constipation in Indonesia and its association with exposure to stressful life events. BMC Gastroenterology 2018;18:146. https://doi.org/10.1186/s12876-018-0873-0.

[6] Joinson C, Grzeda MT, von Gontard A, et al. Psychosocial risks for constipation and soiling in primary school children. Eur Child Adolesc Psychiatry 2019;28:203–10. https://doi.org/10.1007/s00787-018-1162-8.

[7] Sawyer G, Heron J, Joinson C. The relationship between maternal psychopathology and offspring incontinence and constipation at school age: A prospective cohort study. J Affect Disord 2023;335:1–9. https://doi.org/10.1016/j.jad.2023.05.003.

[8] Gozali FS, Febiana B, Putra IGNS, et al. Relationship between psychological stress with functional constipation in children: a systematic review. Pan Afr Med J 2023;46:8. https://doi.org/10.11604/pamj.2023.46.8.41130.

[9] van Dijk M, de Vries G-J, Last BF, et al. Parental child-rearing attitudes are associated with functional constipation in childhood. Arch Dis Child 2015;100:329–33. https://doi.org/10.1136/archdischild-2014-305941.

[10] Taylor CM, Northstone K, Wernimont SM, et al. Picky eating in preschool children: Associations with dietary fibre intakes and stool hardness. Appetite 2016;100:263–71. https://doi.org/10.1016/j.appet.2016.02.021.

[11] Heron J, Grzeda M, Tappin D, et al. Early childhood risk factors for constipation and soiling at school age: an observational cohort study. BMJ Paediatr Open 2018;2:e000230. https://doi.org/10.1136/bmjpo-2017-000230.

[12] Tappin D, Grzeda M, Joinson C, et al. Challenging the view that lack of fibre causes childhood constipation. Arch Dis Child 2020;105:864–8. https://doi.org/10.1136/archdischild-2019-318082.

[13] Wang GN, Zhang K, Xiong YY, et al. The relationship between functional constipation and overweight/obesity in children: a systematic review and meta-analysis. Pediatr Res 2023;94:1878–86. https://doi.org/10.1038/s41390-023-02711-1.

[14] Fraser A, Macdonald-Wallis C, Tilling K, et al. Cohort profile: the Avon Longitudinal Study of Parents and Children: ALSPAC mothers cohort. Int J Epidemiol 2013;42:97–110. https://doi.org/10.1093/ije/dys066.

[15] Boyd A, Golding J, Macleod J, et al. Cohort profile: The ‘Children of the 90s’—the index offspring of the Avon Longitudinal Study of Parents and Children. Int J Epidemiol 2013;42:111–27. https://doi.org/10.1093/ije/dys064.

Confidential: For Review Only

[16] Joinson C, Grzeda MT, von Gontard A, et al. Psychosocial risks for constipation and soiling in primary school children. Eur Child Adolesc Psychiatry 2019;28:203–10. https://doi.org/10.1007/s00787-018-1162-8.
[17] dos Santos IR, de Abreu GE, Dourado ER, et al. Emotional and behavioural problems in children and adolescents: The role of constipation. Journal of Paediatrics and Child Health 2021;57:1003–8. https://doi.org/10.1111/jpc.15368.
[18] Goodman R. Psychometric properties of the strengths and difficulties questionnaire. J Am Acad Child Adolesc Psychiatry 2001;40:1337–45. https://doi.org/10.1097/00004583-200111000-00015.
[19] Robins J. A new approach to causal inference in mortality studies with a sustained exposure period—application to control of the healthy worker survivor effect. Mathematical Modelling 1986;7:1393–512. https://doi.org/10.1016/0270-0255(86)90088-6.
[20] Daniel RM, De Stavola BL, Cousens SN. Gformula: Estimating Causal Effects in the Presence of Time-Varying Confounding or Mediation using the G-Computation Formula. The Stata Journal 2011;11:479–517. https://doi.org/10.1177/1536867X1201100401.
[21] VanderWeele TJ, Vansteelandt S. Odds Ratios for Mediation Analysis for a Dichotomous Outcome. Am J Epidemiol 2010;172:1339–48. https://doi.org/10.1093/aje/kwq332.
[22] StataCorp. Stata Statistical Software: Release 17 2023.
[23] R Core Team. R: A language and environment for statistical computing. 2022.
[24] Royston P, White IR. Multiple Imputation by Chained Equations (MICE): Implementation in Stata. Journal of Statistical Software 2011;45:1–20. https://doi.org/10.18637/jss.v045.i04.
[25] Rubin DB. Multiple Imputation for Nonresponse in Surveys. New York, NY: John Wiley & Sons; 2004.
[26] Edan OAA, Yahya FS. Socio-demographic characteristics and risk factors of functional constipation in children: A case-control study. Sri Lanka Journal of Child Health 2022;51. https://doi.org/10.4038/sljch.v51i2.10123.
[27] Jena A, Montoya CA, Mullaney JA, et al. Gut-Brain Axis in the Early Postnatal Years of Life: A Developmental Perspective. Front Integr Neurosci 2020;14. https://doi.org/10.3389/fnint.2020.00044.
[28] Rhee SH, Pothoulakis C, Mayer EA. Principles and clinical implications of the brain-gut-enteric microbiota axis. Nat Rev Gastroenterol Hepatol 2009;6:306–14. https://doi.org/10.1038/nrgastro.2009.35.
[29] Mota DM, Barros AJD. Toilet training: methods, parental expectations and associated dysfunctions. J Pediatr (Rio J) 2008;84:9–17. https://doi.org/10.2223/JPED.1752.
[30] Malowitz S, Green M, Karpinski A, et al. Age of Onset of Functional Constipation. J Pediatr Gastroenterol Nutr 2016;62:600–2. https://doi.org/10.1097/MPG.0000000000001011.
[31] Blum NJ, Taubman B, Osborne ML. Behavioral characteristics of children with stool toileting refusal. Pediatrics 1997;99:50–3. https://doi.org/10.1542/peds.99.1.50.
[32] Schonwald A, Sherritt L, Stadtler A, et al. Factors associated with difficult toilet training. Pediatrics 2004;113:1753–7. https://doi.org/10.1542/peds.113.6.1753.
[33] Chogle A, Wong G, Megerian JT. Constipation in Children with Autism: A Comprehensive Review. Curr Treat Options Peds 2024;10:287–94. https://doi.org/10.1007/s40746-024-00304-6.

Confidential: For Review Only

[34] Gyamenah P, Burrows K, Rai D, et al. Associations of Autistic Traits and Autism with Incontinence and Constipation in a UK Birth Cohort. J Autism Dev Disord 2024. https://doi.org/10.1007/s10803-024-06663-1.
[35] Shkalim Zemer V, Cohen HA, Richenberg Y, et al. Personal hygiene, environmental conditions, and toilet use of children in primary schools: A cohort study. J Pediatr Urol 2023;19:721–7. https://doi.org/10.1016/j.jpurol.2023.06.004.
[36] Koppen IJN, von Gontard A, Chase J, et al. Management of functional nonretentive fecal incontinence in children: Recommendations from the International Children's Continence Society. J Pediatr Urol 2016;12:56–64. https://doi.org/10.1016/j.jpurol.2015.09.008.
[37] Whale K, Cramer H, Joinson C. Left behind and left out: The impact of the school environment on young people with continence problems. Br J Health Psychol 2018;23:253–77. https://doi.org/10.1111/bjhp.12284.
[38] Sterne JAC, White IR, Carlin JB, et al. Multiple imputation for missing data in epidemiological and clinical research: potential and pitfalls. BMJ 2009;338:b2393–b2393. https://doi.org/10.1136/bmj.b2393.
[39] Mugie SM, Benninga MA, Di Lorenzo C. Epidemiology of constipation in children and adults: A systematic review. Best Practice & Research Clinical Gastroenterology 2011;25:3–18. https://doi.org/10.1016/j.bpg.2010.12.010.
[40] Vriesman MH, Rajindrajith S, Koppen IJN, et al. Quality of Life in Children with Functional Constipation: A Systematic Review and Meta-Analysis. J Pediatr 2019;214:141–50. https://doi.org/10.1016/j.jpeds.2019.06.059.

FIGURES

Figure 1 Flow chart of inclusion and exclusion of ALSPAC participants into the study sample

ALSPAC Avon longitudinal study of parents and children; FAI family adversity index. Mediators include emotional/behaviour problems and constipation. Confounders include sex, ethnicity, preterm birth, household social class, marital status, and smoking during pregnancy. Intermediate confounders include child developmental delay, maternal bond score, parental abuse, stool frequency, hard stools, difficult temperament, picky eating, healthy eating, temper tantrums, and BMI.

Figure 2 Directed Acyclic Graph (DAG) illustrating hypothesised causal relationships between the family adversity index, mediators and daytime soiling

Directed acyclic graph (DAG) of the mediation model. Each intermediate confounding path was specified separately but are grouped together for clarity. For simplicity baseline confounders are omitted from the DAG as all confounders are proposed to cause all other model variables.

Confidential: For Review Only

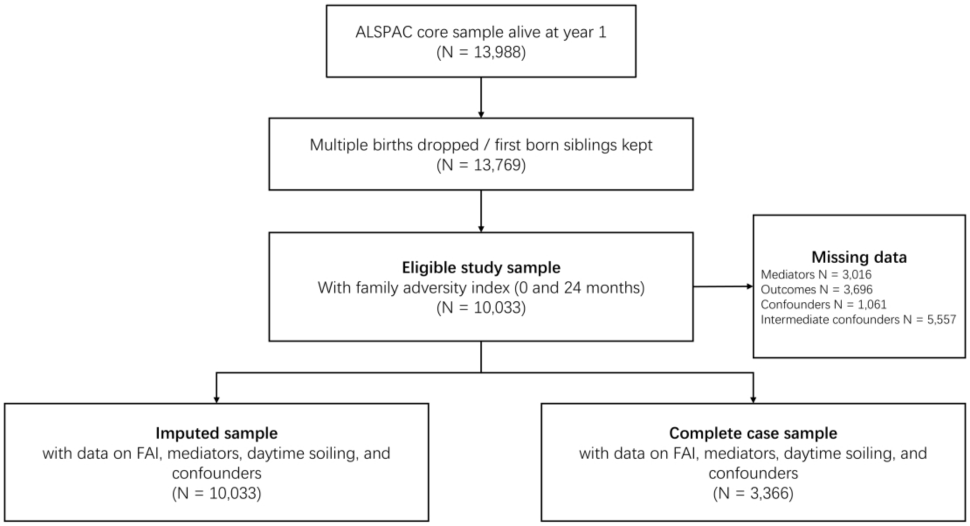

ALSPAC Avon longitudinal study of parents and children; FAI family adversity index. Mediators include emotional/behaviour problems and constipation. Confounders include sex, ethnicity, preterm birth, household social class, marital status, and smoking during pregnancy. Intermediate confounders include child developmental delay, maternal bond score, parental abuse, stool frequency, hard stools, difficult temperament, picky eating, healthy eating, temper tantrums, and BMI.

338x190mm (300 x 300 DPI)

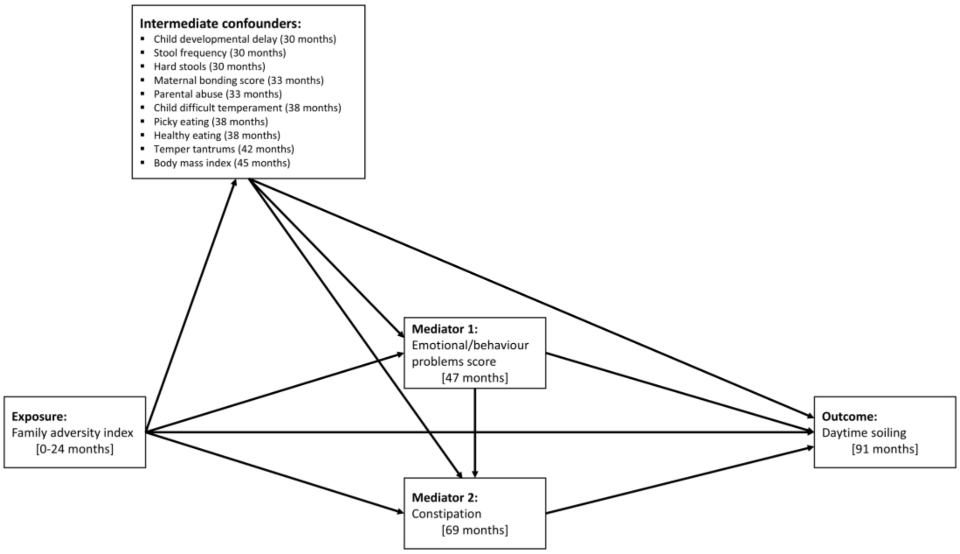

Directed acyclic graph (DAG) of the mediation model. Each intermediate confounding path was specified separately but are grouped together for clarity. For simplicity baseline confounders are omitted from the DAG as all confounders are proposed to cause all other model variables.

338x190mm (300 x 300 DPI)

Examining causal pathways between family adversity and soiling: a prospective cohort study

Contents

Supplementary Text..2
Methods: Sample 2
Methods: The Family Adversity Index (FAI)..2
Methods: The parametric g-formula..3
Methods: Missing data 3
Results: Description of sample...4
Supplementary Tables..5
Table S1 Variable description and coding 5
Table S2 Auxiliary variables for multiple imputation of missing data 10
Table S3 Prevalences of the individual adversity components of the family adversity index in (N = 10,033)..11
Table S4 Results for the associations between family adversity index and mediators in the imputed data sample (N = 10,033)...12
Table S5 Results for the associations between emotional/behaviour problems and constipation in the imputed data sample (N = 10,033)..12
Table S6 Results for the associations between family adversity index and mediators with daytime soiling in the complete case sample (N = 3,366) 12
Table S7 Results for the associations between family adversity index and mediators in the complete case sample (N = 3,366) 13
Table S8 Results for the mediation of family adversity index and daytime soiling via emotional/behaviour problems and constipation in the complete case sample (N = 3,366)..13
References 14
Strobe statement 15

Confidential: For Review Only

Supplementary Text

Methods: Sample

ALSPAC is a prospective, population-based birth cohort study that recruited pregnant women resident in Avon, UK with expected dates of delivery between 1st April 1991 and 31st December 1992 [1,2]. The initial number of pregnancies enrolled was 14,541 resulting in 14,062 live births and 13,988 children who were alive at 1 year of age. When the oldest children were approximately 7 years of age, an attempt was made to bolster the initial sample with eligible cases who had failed to join the study originally, resulting in an additional 913 children being enrolled. The total sample size for analyses using any data collected after the age of seven is therefore 15 447 pregnancies, resulting in 15,658 foetuses. Of these 14,901 children were alive at 1 year of age. 14,203 unique mothers were initially enrolled in the study and 12,113 partners have been in contact with the study of which 3,807 are enrolled. The phases of enrolment are described in more detail in the cohort profile paper and its update [1,2]. The study website (www.bristol.ac.uk/alspac) contains details of all the data that is available through a fully searchable data dictionary and variable search tool: http://www.bristol.ac.uk/alspac/researchers/our-data/. Ethical approval for ALSPAC was obtained from the ALSPAC Ethics and Law Committee and the Local Research Ethics Committees. Informed consent for the use of data collected via questionnaires and clinics was obtained from participants following the recommendations of the ALSPAC Ethics and Law Committee at the time.

Methods: The Family Adversity Index (FAI)

The FAI was derived from responses to questions asked about ALSPAC participants (parents and children) that reflected the following family-based risk factors:

- **Age of mother*** (1): at first pregnancy/child's birth
- **Housing** (3): a) adequacy, b) basic living, c) defects/infestation
- **No educational qualifications*** (1) achievement (mother or father)
- **Financial status** (1): a) financial difficulties
- **Partner relationship** (4): a) status, b) affection and aggression, c) physical/emotional cruelty, d) no social support
- **Family** (2): a) family size, b) Major problems (child in care, not with natural mother, or on Social Services at-risk register**)
- **Social network** (2): a) emotional support, b) Practical/financial support
- **Maternal Psychopathology** (1): a) Affective (depression, anxiety, or suicide attempts)
- **Substance abuse** (1): a) drugs or alcohol (use of hard drugs, alcoholism, alcohol consumption)
- **Crime** (2): a) In trouble with police, or b) convictions

*[The numbers in brackets refer to the number of items within each category. *Note that these items were measured during the antenatal period or at birth. **The at-risk register has been superseded by the Child Protection Plan (CPP); The Children Act 1989]*

The FAI scores were calculated where >50% of the above items were valid and non-adversity was assumed for any missing data. Each item was assigned a value of 1 if the adversity was present and 0 if not. The final score was derived from the sum of

items giving a possible score range from 0 to 18 and the higher the score recorded the greater the level of adversity.

Details of the questionnaires from which these items were derived are available in the ALSPAC study documentation file D4297_mLon.pdf held in the publicly available data dictionary:

https://www.bristol.ac.uk/alspac/external/documents/ALSPAC_Data_Dictionary.zip

<u>Methods: The parametric g-formula</u>

In mediation analysis, four assumptions are made with respect to confounding: no unmeasured confounders for the exposure-outcome, exposure-mediator or mediator-outcome paths; no measured or unmeasured confounders of the mediator-outcome path that lie on the causal pathway from the exposure. We implemented the parametric g-computation approach, which relaxes the assumption of no measured intermediate confounders [3,4].

We used the Stata package *gformula* to conduct parametric g-computation to estimate the total causal effect (TCE), natural direct effect (NDE) and the natural indirect effect (NIE) [3]. G-formula uses Monte Carlo simulations to simulate the outcome, mediators and intermediate confounders under hypothetical interventions or “counter to the fact” scenarios (i.e. contrary to the intervention individuals actually received). The TCE is the value the outcome (e.g. daytime soiling) would take if all individuals had been exposed to a one-unit increase in the FAI versus everyone not being exposed to a one unit increase in the FAI. The NDE is the direct (unmediated) effect of the exposure (e.g. FAI) on the outcome (e.g. daytime soiling) when the mediator(s) (e.g. constipation) takes the value it would be in absence of the exposure (i.e. FAI of 0). More specifically it is modelled as the direct effect of exposure X = x+1 (e.g. an FAI of 1) versus exposure X = x (e.g. an FAI of 0) on outcome Y (e.g. daytime soiling) if mediator M (e.g. constipation) were set to whatever value it would be for X = x. The NIE is the effect of the exposure (e.g. FAI) on the outcome (e.g. daytime soiling) that operates by changing the mediator (e.g. constipation). More specifically, it is modelled as the effect on outcome Y (e.g. daytime soiling) if the exposure X = x+1 and the mediator M (e.g. constipation) were changed from the value it would take if X = x to the value it would take if X = x+1. The proportion of the TCE that is mediated is calculated as $[OR_{NDE} (OR_{NIE} - 1)] / [OR_{NDE} \times OR_{NIE} - 1] \times 100$ [5].

Specifically for this analysis we used a Monte Carlo sample size of 100,000 to minimise fluctuations in effect estimates. Standard errors were estimated using 250 bootstrap samples. We did not hypothesise an interaction between the exposure and mediators and therefore did not include an interaction term within the model. We also specified the command option *linexp* to specify that the exposure is continuous, and its effect is assumed to be linear. For the imputed data we conducted mediation analysis for each of the 50 imputation datasets and output the results to log files. We used R to extract the log ORs and bootstrapped SEs for each of the TCE, NDE, and NIE from each Stata log file. The mean of the log OR was computed and the SEs were calculated using Rubin’s rules [6]. The 95% confidence intervals were calculated from the combined log OR and SEs. Log ORs (and 95% CIs) were exponentiated to represent ORs.

<u>Methods: Missing data</u>

We used multivariate imputation by chained equations under the missing at random assumption to impute missing data [7] in outcome daytime soiling and baseline and

Confidential: For Review Only

intermediate confounders up to the sample with complete data on the exposure FAI score (N = 10,033). We included two types of auxiliary variables; i) additional measures (both before and/or after) of those of the substantive model, and ii) sociodemographic indicators associated with both missingness and many of the variables of the substantive model. These serve two main functions: 1) increasing precision by improving the predictive ability of the imputation model beyond that of the substantive model [8] and 2) making the MAR assumption more plausible by reducing the bias due to data being MNAR [9] (see Supplementary Table S2). All binary variables were imputed using logistic regression, categorical (unordered) variables were imputed using polytomous regression, and all continuous variables were imputed using predictive mean matching. Missing data were imputed separately in males and females before appending the two datasets together. For both males and females 50 imputed datasets with 50 iterations were created using the mice package (version 3.16.0) in R. Estimates were then combined using Rubin's rules [6].

Results: Description of sample

Table 1 in the main manuscript shows the distribution of study variables in the imputed (N = 10 033) and complete case samples (N = 3366). Any daytime soiling was reported for 7.3% of children. In total, 71.7% of children experienced one or more family adversities, the most prevalent being maternal affective disorders (depression, anxiety, and/or suicide attempt; 20.5%). A lack of social support from partner relationships was also common (19.6%; see Supplementary Table 3 for all component prevalences). The mean number of family adversities experienced was 1.9 (Standard Error (SE) 0.02) in all children and was higher in those experiencing soiling (2.3, SE 0.10) compared to those without soiling (1.8, SE 0.02; ttest p-value <0.001). Compared to the imputed data sample, children in the complete case sample experienced fewer adversities and lower proportions of non-white ethnicity, manual social class, preterm birth, parental abuse, and temper tantrums. The complete case sample also had a higher proportion of children with picky eating, hard stools, no maternal smoking during pregnancy, and maternal married status.

Confidential: For Review Only

Supplementary Tables

Table S1 Variable description and coding

Variable name	ALSPAC variable[1]	Time of measure	Measure/Question	Responses	Final variable coding
Exposure					
**Family Adversity Index (FAI)**<br>From 0-2 years	blong	0 to 2 years	DV: long index comprising of 18 items including information on the age of the mother, housing, education, financial status, partner relationship, family, social network, maternal psychopathology, substance abuse and crime	Continuous score (0-18)	Continuous score (0-18)
Outcome					
**Daytime soiling**<br>Frequency child dirties pants during the day	kr125	7.6 years	How often usually does your child dirty his/her pants during the day?	1: Never<br>2: < once /week<br>3: ~ once / week<br>4: 2-5 times / week<br>5: Nearly every day<br>6: > once / day<br>-6; -1: Missing	Recode:<br>1=0: No<br>2/6=1: Yes
Mediators					
**Emotional/behaviour problems**<br>Parent version of the Strengths and Difficulties Questionnaire by Goodman (1997)<br>Total difficulties score	j557f	3.9 years	Derived variable: prorated score of the first 4 subscales (hyperactivity, conduct problems, emotional problems, and peer problems)	Continuous; missing = -1	Continuous (score 0-33)

Confidential: For Review Only

Variable name	ALSPAC variable[1]	Time of measure	Measure/Question	Responses	Final variable coding
**Constipation**	kn1037	5.8 years	In the past 15 months child has had constipation?	1: Yes & Saw Doctor<br>2: Yes But Did Not See Doctor<br>3: Did Not Have<br>-11; -10; -1: Missing	Recode:<br>3=0: No<br>1/2=1: Yes
Baseline Confounders					
**Ethnicity**<br>Child ethnic background	c804	Antenatal	Derived variable: Ethnic group reported for the mother (respondent) or partner:<br>Non-white if c801 or c802 had codes in the range of 2-9	1: White<br>2: Non-white<br>-1: Missing	Recode:<br>1=0: White<br>2=1: Non-white
**Sex**<br>Child's sex assigned at birth	kz021	Birth	Recorded at birth by the fieldworkers who visited the maternity units	1: Male<br>2: Female<br>-2; -1: Missing	Recode:<br>1=0: Male<br>2=1: Female
**Preterm birth**<br>Term vs. preterm	bestgest	Birth	Derived variable: Best guess of gestation when pregnancy ended	Continuous (range 24-47)<br>-11; -10; -3; -2: Missing	Recode:<br>>36 weeks=0: Term<br>≤36 weeks=1: Preterm
**Marital status**	a525	Antenatal	What is your present marital status?	1: Never married<br>2: Widowed<br>3: Divorced<br>4: Separated<br>5: 1st marriage<br>6: Marriage 2 or 3<br>-1: Missing	Recode:<br>5/6=0: Married<br>1=1: Single<br>2/4=2: Divorced/separated/widowed
**Smoking**<br>Maternal smoking during pregnancy	b665 (1st trimester)	Antenatal	Did you smoke regularly in the first three months of pregnancy?	1: No<br>2: Yes, cigarettes<br>3: Yes, cigars<br>5: Yes, other	Recode:<br>[No (0) smoking during trimesters 1, 2 and 3]=0: None<br>[Smoked during 1st trimester (2, 3, 5) but not the 2nd (0) and 3rd (0)]=1: Yes, quit

Confidential: For Review Only

Variable name	ALSPAC variable[1]	Time of measure	Measure/Question	Responses	Final variable coding
	b667 (2nd trimester)	Antenatal	Did you smoke regularly in the last 2 weeks?	1: No<br>2: Yes, cigarettes<br>3: Yes, cigars<br>5: Yes, other	early<br>[Smoked during trimesters 1 (2, 3, 5), 2 (2, 3, 5), and 3 (1, 2, 3) or either 1 and 2 or 1 and 3, or 2 and 3]=2: Yes, throughout
	c483 (3rd trimester)	Antenatal	How many cigarettes per day are you yourself smoking at the moment?	0: None<br>1: 1-9<br>2: 10-19<br>3: 20+<br>-7: Missing	
**Household social class**<br>1991 British Office of Population and Census Statistics (OPCS) job codes (maternal)<br>1991 British Office of Population and Census Statistics (OPCS) job codes (paternal)	c755<br>c765	Antenatal	Derived variable from questions:<br>*Actual job, occupation, trade or profession<br>*Please tick which of the following apply to you: foreman, manager, supervisor, leading hand, self-employed, none of these<br>*Type of industry or service given (main things done in job)	1 - I<br>2 - II<br>3 – III (non-manual)<br>4 – III (manual)<br>5 - IV<br>6 - V<br>65 - Armed forces<br>-1 - missing	Highest parental social class i.e. if one parent is non-manual then social class=0:<br>[Professional, managerial or skilled professions (responses 1, 2, 3)]=0: Non-manual<br>[Partly or unskilled occupations (responses 4, 5, 6)]=1: Manual<br>65=missing
Intermediate Confounders					
**Body mass index (BMI)**	bmi_chdb4		Child health database measures of BMI: Derived from measure of height (m) and weight (kg).<br>Calculated as kg/m2	Continuous measure	Continuous measure
**Difficult temperament**<br>Emotionality, Activity, and Sociability (EAS) Scale	kg620b	3.2 years	Derived variable: EAS emotionality subscale (prorated)	Continuous (score 5-25)<br>-6; -5: Missing	Continuous (score 5-25)

Confidential: For Review Only

Variable name	ALSPAC variable[1]	Time of measure	Measure/Question	Responses	Final variable coding
**Temper tantrums**<br>Frequency child has temper tantrums	kj255	3.5 years	How often does he/she have temper tantrums?	1: Once per Day<br>2: Most Days<br>3: > Once a WK<br>4: < Once a WK<br>5: Never<br>-1: Missing	Recode:<br>3/4/5=0: Few or no temper tantrums<br>1/2=1: Once a day or most days
**Developmental delay**<br>Total developmental score	kf607	2.5 years	Derived variable: prorated scores	Continuous (age-adjusted z-score); missing = -102; -101	Continuous (z score)
**Hard stools**<br>Frequency child's stools are hard	kf141	2.5 years	Nowadays how often are his/her stools hard?	1: Usually<br>2. Sometimes<br>3. Never<br>-1: Missing	Recode:<br>2/3=0: No<br>1=1: Yes
**Stool frequency**<br>Number of motions child has everyday	kf140	2.5 years	Nowadays how many motions (or dirty nappies) a day (24 hours) does he usually have?	0: Other<br>1: 4 or > 4 times<br>2: 2 - 3 times<br>3: once a day<br>4: once in 2 - 4 days<br>5: once a week<br>9: can't say<br>-1: missing	Recode:<br>1/2/3=0: Frequent<br>4/5=1: Infrequent<br>9=missing
**Picky eating**<br>Likes and dislikes of food	kg466	3.2 years	Does your child have definite likes and dislikes as far as food is concerned?	1: No eats most food<br>2. Yes quite choosy<br>3. Yes very choosy<br>-1: Missing	Recode:<br>1/2=0: No<br>3 =1: Yes, picky eater
**Diet: Fibre intake**<br>PC: Healthy eating (proxy for fibre)<br>Northstone et al 2000	kg521	3.2 years	Derived variable: PCA score 2 'healthy' 38 months	Continuous	Continuous
**Maternal bond**<br>Maternal bonding score	h766	2.8 years	Derived variable from h760 + h763	Continuous score (0-33)<br>-1: Missing	Continuous score (0-33)

Confidential: For Review Only

Variable name	ALSPAC variable[1]	Time of measure	Measure/Question	Responses	Final variable coding
**Parental abuse**<br>Parental cruelty (physical and emotional)<br>Question: Have any of these occurred since the study child was 18 months old?	h236a	2.8 years	Partner physically cruel to children	1: Yes<br>2: No<br>-1: Missing	Recode:<br>[h236a, h237a, h247a, h248a: 2]=0: No<br>[any h236a, h237a, h247a, h248a: 1]=1: Yes<br>For imputation derive:<br>Physical cruelty [h236a, h237a: 2]=0: No / [h236a, h237a: 1]=1: Yes<br>Emotional cruelty [h247a, h248a: 2]=0: No / [h247a, h248a: 1]=1: Yes<br>Then passively impute:<br>Physical cruelty = No & Emotional cruelty = No ==0: No parental abuse<br>Physical cruelty = Yes &/or Emotional cruelty = Yes ==1: Yes parental abuse
	h237a	2.8 years	Mother physically cruel to children	1: Yes<br>2: No<br>-1: Missing	
	h247a	2.8 years	Partner emotionally cruel to children	1: Yes<br>2: No<br>-1: Missing	
	h248a	2.8 years	Mother emotionally cruel to children	1: Yes<br>2: No<br>-1: Missing	

[1] ALSPAC variable names; these can be searched for using the ALSPAC variable search tool [http://variables.alspac.bris.ac.uk/].

Confidential: For Review Only

Table S2 Auxiliary variables for multiple imputation of missing data

Daytime soiling in child measured at age 6
Daytime soiling in child measured at age 6
Total difficulties score measured at age 6
Constipation in child measured at age 8
Stool frequency measured at age 3
Hard stools measured at age 3
BMI measured at age 18 months
Picky eating measured at age 2
Healthy eating measured at age 2
House tenure measured in antenatal period
Household crowding measured in antenatal period
Material hardship measured in antenatal period
Antenatal depression in mothers measured in antenatal period
Antenatal anxiety in mothers measured in antenatal period

Confidential: For Review Only

Table S3 Prevalences of the individual adversity components of the family adversity index in (N = 10,033)

FAI constituent	Sub-measure affective group	Prevalence (%)
**Age of mother**	Early parenthood	5.2
**Crime**	Been in trouble with police	3.7
**Crime**	Has convictions	0.6
**Family**	High family size	5.5
**Family**	Has family major problems	0.9
**Financial status**	financial difficulties	15.3
**Housing**	Has housing defects/infestation	17.5
**Housing**	Poor housing adequacy	8.6
**Housing**	Poor housing - basic living	4.8
**Maternal psychopathology**	Has affective disorders (depression, anxiety and/or suicide attempts)	20.5
**No educational qualifications**	Low educational achievement (mother or father)	12.4
**Partner relationship**	Lack of partner social support	19.6
**Partner relationship**	Lack of partner affection	14.7
**Partner relationship**	Experienced partner cruelty	12.9
**Partner relationship**	Partner relationship status	6.5
**Social network**	Lack of emotional support	12.6
**Social network**	Lack of practical/financial support	11.2
**Substance abuse**	Substance/alcohol abuse	13.4

FAI Family adversity index

Confidential: For Review Only

Table S4 Results for the associations between family adversity index and mediators in the imputed data sample (N = 10,033)

Exposure: FAI		Univariable			Multivariable		
		Beta/OR	95% CI	p-value	Beta/OR	95% CI	p-value
Outcome	Emotional/behaviour problems	0.587	0.537, 0.638	<0.001	0.524	0.471, 0.577	<0.001
	Constipation	1.086	1.047, 1.125	<0.001	1.072	1.033, 1.113	<0.001

OR odds ratio; CI confidence interval; FAI family adversity index; all multivariable models adjusted for baseline confounders (sex, ethnicity, preterm birth, household social class, marital status, and smoking during pregnancy). Parameter estimates for emotional/behaviour problems are Betas; constipation are ORs.

Table S5 Results for the associations between emotional/behaviour problems and constipation in the imputed data sample (N = 10,033)

Exposure: Emotional/behaviour problems	Univariable			Multivariable		
	OR	95% CI	p-value	OR	95% CI	p-value
Constipation	1.033	1.017, 1.050	<0.001	1.007	0.986, 1.027	0.521

OR odds ratio; CI confidence interval; multivariable models adjusted for baseline confounders (sex, ethnicity, preterm birth, household social class, marital status, and smoking during pregnancy) and the Family Adversity Index.

Table S6 Results for the associations between family adversity index and mediators with daytime soiling in the complete case sample (N = 3,366)

Outcome: daytime soiling		Univariable			Multivariable		
		OR	95% CI	p-value	OR	95% CI	p-value
Exposure	FAI	1.080	0.998, 1.165	0.051	1.104	1.015, 1.196	0.019
	Emotional/behaviour problems[1]	1.029	0.999, 1.061	0.059	0.995	0.959, 1.032	0.805
	Constipation[1,2]	3.309	2.355, 4.591	<0.001	3.596	2.525, 5.063	<0.001

OR odds ratio; CI confidence interval; FAI family adversity index; all multivariable models adjusted for baseline confounders (sex, ethnicity, preterm birth, household social class, marital status, and smoking during pregnancy). [1] Models additionally adjusted for intermediate confounders (child developmental delay, maternal bonding score, parental abuse, stool frequency, hard stools, difficult temperament, picky eating, healthy eating, temper tantrums, and body mass index), and FAI. [2] Model additionally adjusted for emotional/behaviour problems. Parameter estimates are based on a one-unit increase in FAI, a one-unit increase in emotional/behaviour problems score, and the comparison between yes vs. no for constipation.

Confidential: For Review Only

Table S7 Results for the associations between family adversity index and mediators in the complete case sample (N = 3,366)

	Exposure: FAI	Univariable			Multivariable		
		Beta/OR	95% CI	p-value	Beta/OR	95% CI	p-value
Outcome	Emotional/behaviour problems	0.625	0.534, 0.717	<0.001	0.585	0.490, 0.680	<0.001
	Constipation	1.100	1.031, 1.171	0.003	1.086	1.015, 1.160	0.015

OR odds ratio; CI confidence interval; FAI family adversity index; all multivariable models adjusted for baseline confounders (sex, ethnicity, preterm birth, household social class, marital status, and smoking during pregnancy). Parameter estimates for emotional/behaviour problems are Betas; constipation are ORs.

Table S8 Results for the mediation of family adversity index and daytime soiling via emotional/behaviour problems and constipation in the complete case sample (N = 3,366)

Total causal effect		Natural indirect effect		Natural direct effect		PM%
OR	95% CI	OR	95% CI	OR	95% CI	
1.092	1.007, 1.215	0.997	0.957, 1.017	1.096	1.023, 1.017	NA

OR odds ratio; CI confidence interval; PM proportion mediated (%). Mediation models were fitted with emotional/behaviour problems and constipation as the mediators of interest; child developmental delay, maternal bonding score, parental abuse, stool frequency, hard stools, difficult temperament, picky eating, healthy eating, temper tantrums, and body mass index were considered intermediate confounders. All paths were adjusted for baseline confounders: sex, ethnicity, preterm birth, household social class, marital status, and smoking during pregnancy.

Confidential: For Review Only

References

[1] Fraser A, Macdonald-Wallis C, Tilling K, et al. Cohort profile: the Avon Longitudinal Study of Parents and Children: ALSPAC mothers cohort. Int J Epidemiol 2013;42:97–110. https://doi.org/10.1093/ije/dys066.

[2] Boyd A, Golding J, Macleod J, et al. Cohort profile: The 'Children of the 90s'—the index offspring of the Avon Longitudinal Study of Parents and Children. Int J Epidemiol 2013;42:111–27. https://doi.org/10.1093/ije/dys064.

[3] Robins J. A new approach to causal inference in mortality studies with a sustained exposure period—application to control of the healthy worker survivor effect. Mathematical Modelling 1986;7:1393–512. https://doi.org/10.1016/0270-0255(86)90088-6.

[4] De Stavola BL, Daniel RM, Ploubidis GB, et al. Mediation Analysis With Intermediate Confounding: Structural Equation Modeling Viewed Through the Causal Inference Lens. Am J Epidemiol 2015;181:64–80. https://doi.org/10.1093/aje/kwu239.

[5] VanderWeele TJ, Vansteelandt S. Odds Ratios for Mediation Analysis for a Dichotomous Outcome. Am J Epidemiol 2010;172:1339–48. https://doi.org/10.1093/aje/kwq332.

[6] Rubin DB. Multiple Imputation for Nonresponse in Surveys. John Wiley & Sons; 2004.

[7] Royston P, White IR. Multiple Imputation by Chained Equations (MICE): Implementation in Stata. Journal of Statistical Software 2011;45:1–20. https://doi.org/10.18637/jss.v045.i04.

[8] Collins LM, Schafer JL, Kam CM. A comparison of inclusive and restrictive strategies in modern missing data procedures. Psychol Methods 2001;6:330–51.

[9] Cornish RP, Macleod J, Carpenter JR, et al. Multiple imputation using linked proxy outcome data resulted in important bias reduction and efficiency gains: a simulation study. Emerg Themes Epidemiol 2017;14:14. https://doi.org/10.1186/s12982-017-0068-0.

Confidential: For Review Only

Strobe statement

STROBE Statement—checklist of items that should be included in reports of observational studies

	Item No	Recommendation
**Title and abstract** **See title and abstract**	1	(*a*) Indicate the study's design with a commonly used term in the title or the abstract
		(*b*) Provide in the abstract an informative and balanced summary of what was done and what was found
**Introduction**		
Background/rationale **Introduction paragraphs 1 & 2**	2	Explain the scientific background and rationale for the investigation being reported
Objectives **Introduction paragraph 3, Figure 2**	3	State specific objectives, including any prespecified hypotheses
**Methods**		
Study design **Methods**	4	Present key elements of study design early in the paper
Setting **Methods: Sample, Supplementary Text**	5	Describe the setting, locations, and relevant dates, including periods of recruitment, exposure, follow-up, and data collection
Participants **Methods: sample, Figure 1, Supplementary Text**	6	(*a*) *Cohort study*—Give the eligibility criteria, and the sources and methods of selection of participants. Describe methods of follow-up<br>*Case-control study*—Give the eligibility criteria, and the sources and methods of case ascertainment and control selection. Give the rationale for the choice of cases and controls<br>*Cross-sectional study*—Give the eligibility criteria, and the sources and methods of selection of participants
		(*b*) *Cohort study*—For matched studies, give matching criteria and number of exposed and unexposed<br>*Case-control study*—For matched studies, give matching criteria and the number of controls per case
Variables **Methods, Supplementary Tables S1 & S2**	7	Clearly define all outcomes, exposures, predictors, potential confounders, and effect modifiers. Give diagnostic criteria, if applicable
Data sources/ measurement **Methods, Supplementary Tables S1, Supplementary Text**	8*	For each variable of interest, give sources of data and details of methods of assessment (measurement). Describe comparability of assessment methods if there is more than one group
Bias **Methods: Statistical analysis and missing data, Supplementary Text, Discussion**	9	Describe any efforts to address potential sources of bias
Study size	10	Explain how the study size was arrived at

Confidential: For Review Only

**Methods: Sample, Figure 1, Supplementary Text**		
Quantitative variables **Methods: Statistical analysis, Supplementary Table S1**	11	Explain how quantitative variables were handled in the analyses. If applicable, describe which groupings were chosen and why
Statistical methods **Methods: Statistical analysis, Supplementary Text**	12	(*a*) Describe all statistical methods, including those used to control for confounding
		(*b*) Describe any methods used to examine subgroups and interactions
		(*c*) Explain how missing data were addressed
		(*d*) *Cohort study*—If applicable, explain how loss to follow-up was addressed<br>*Case-control study*—If applicable, explain how matching of cases and controls was addressed<br>*Cross-sectional study*—If applicable, describe analytical methods taking account of sampling strategy
		(*e*) Describe any sensitivity analyses
**Results**		
Participants **Figure 1, Supplementary Text, Table 1**	13*	(a) Report numbers of individuals at each stage of study—eg numbers potentially eligible, examined for eligibility, confirmed eligible, included in the study, completing follow-up, and analysed
		(b) Give reasons for non-participation at each stage
		(c) Consider use of a flow diagram
Descriptive data **Table 1, Results**	14*	(a) Give characteristics of study participants (eg demographic, clinical, social) and information on exposures and potential confounders
		(b) Indicate number of participants with missing data for each variable of interest
		(c) *Cohort study*—Summarise follow-up time (eg, average and total amount)
Outcome data **Table 1, Results**	15*	*Cohort study*—Report numbers of outcome events or summary measures over time
		Case-control study—Report numbers in each exposure category, or summary measures of exposure
		Cross-sectional study—Report numbers of outcome events or summary measures
Main results **Results, Tables 1-3, Supplementary Tables S3-7**	16	(*a*) Give unadjusted estimates and, if applicable, confounder-adjusted estimates and their precision (eg, 95% confidence interval). Make clear which confounders were adjusted for and why they were included
		(*b*) Report category boundaries when continuous variables were categorized
		(*c*) If relevant, consider translating estimates of relative risk into absolute risk for a meaningful time period
Other analyses **Complete case analysis Supplementary Tables S5-7**	17	Report other analyses done—eg analyses of subgroups and interactions, and sensitivity analyses
**Discussion**		

Confidential: For Review Only

Key results<br>**Discussion paragraph 1**	18	Summarise key results with reference to study objectives
Limitations<br>**Discussion: Strengths and limitations**	19	Discuss limitations of the study, taking into account sources of potential bias or imprecision. Discuss both direction and magnitude of any potential bias
Interpretation<br>**Discussion: Comparison with previous findings and potential mechanisms**	20	Give a cautious overall interpretation of results considering objectives, limitations, multiplicity of analyses, results from similar studies, and other relevant evidence
Generalisability<br>**Discussion: Strengths and limitations**	21	Discuss the generalisability (external validity) of the study results
**Other information**		
Funding<br>**Funding section**	22	Give the source of funding and the role of the funders for the present study and, if applicable, for the original study on which the present article is based

*Give information separately for cases and controls in case-control studies and, if applicable, for exposed and unexposed groups in cohort and cross-sectional studies.

Note: An Explanation and Elaboration article discusses each checklist item and gives methodological background and published examples of transparent reporting. The STROBE checklist is best used in conjunction with this article (freely available on the Web sites of PLoS Medicine at http://www.plosmedicine.org/, Annals of Internal Medicine at http://www.annals.org/, and Epidemiology at http://www.epidem.com/). Information on the STROBE Initiative is available at www.strobe-statement.org.

Confidential: For Review Only
